# The missing part: the *Archaeoglobus fulgidus* Argonaute forms a functional heterodimer with an N-L1-L2 domain protein

**DOI:** 10.1093/nar/gkad1241

**Published:** 2024-01-09

**Authors:** Elena Manakova, Edvardas Golovinas, Reda Pocevičiūtė, Giedrius Sasnauskas, Arunas Silanskas, Danielis Rutkauskas, Marija Jankunec, Evelina Zagorskaitė, Edvinas Jurgelaitis, Algirdas Grybauskas, Česlovas Venclovas, Mindaugas Zaremba

**Affiliations:** Institute of Biotechnology, Life Sciences Center, Vilnius University, Sauletekio av. 7, LT-10257, Vilnius, Lithuania; Institute of Biotechnology, Life Sciences Center, Vilnius University, Sauletekio av. 7, LT-10257, Vilnius, Lithuania; Institute of Biotechnology, Life Sciences Center, Vilnius University, Sauletekio av. 7, LT-10257, Vilnius, Lithuania; Institute of Biotechnology, Life Sciences Center, Vilnius University, Sauletekio av. 7, LT-10257, Vilnius, Lithuania; Institute of Biotechnology, Life Sciences Center, Vilnius University, Sauletekio av. 7, LT-10257, Vilnius, Lithuania; Institute of Biotechnology, Life Sciences Center, Vilnius University, Sauletekio av. 7, LT-10257, Vilnius, Lithuania; Institute of Physics, Center for Physical Sciences and Technology, Savanoriu 231, LT-02300, Vilnius, Lithuania; Institute of Biotechnology, Life Sciences Center, Vilnius University, Sauletekio av. 7, LT-10257, Vilnius, Lithuania; Institute of Biochemistry, Life Sciences Center, Vilnius University, Sauletekio av. 7, LT-10257, Vilnius, Lithuania; Institute of Biotechnology, Life Sciences Center, Vilnius University, Sauletekio av. 7, LT-10257, Vilnius, Lithuania; Institute of Biotechnology, Life Sciences Center, Vilnius University, Sauletekio av. 7, LT-10257, Vilnius, Lithuania; Institute of Biotechnology, Life Sciences Center, Vilnius University, Sauletekio av. 7, LT-10257, Vilnius, Lithuania; Institute of Biotechnology, Life Sciences Center, Vilnius University, Sauletekio av. 7, LT-10257, Vilnius, Lithuania; Institute of Biotechnology, Life Sciences Center, Vilnius University, Sauletekio av. 7, LT-10257, Vilnius, Lithuania

## Abstract

Argonaute (Ago) proteins are present in all three domains of life (bacteria, archaea and eukaryotes). They use small (15–30 nucleotides) oligonucleotide guides to bind complementary nucleic acid targets and are responsible for gene expression regulation, mobile genome element silencing, and defence against viruses or plasmids. According to their domain organization, Agos are divided into long and short Agos. Long Agos found in prokaryotes (long-A and long-B pAgos) and eukaryotes (eAgos) comprise four major functional domains (N, PAZ, MID and PIWI) and two structural linker domains L1 and L2. The majority (∼60%) of pAgos are short pAgos, containing only the MID and inactive PIWI domains. Here we focus on the prokaryotic Argonaute AfAgo from *Archaeoglobus fulgidus* DSM4304. Although phylogenetically classified as a long-B pAgo, AfAgo contains only MID and catalytically inactive PIWI domains, akin to short pAgos. We show that AfAgo forms a heterodimeric complex with a protein encoded upstream in the same operon, which is a structural equivalent of the N-L1-L2 domains of long pAgos. This complex, structurally equivalent to a long PAZ-less pAgo, outperforms standalone AfAgo in guide RNA-mediated target DNA binding. Our findings provide a missing piece to one of the first and the most studied pAgos.

## Graphical Abstract

**Graphical Abstract:**
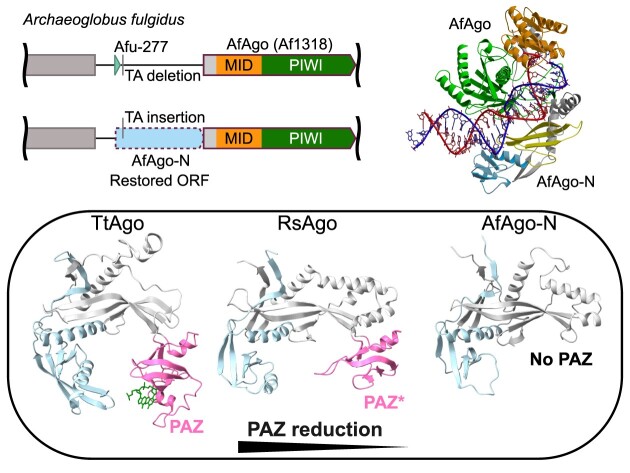

## Introduction

Argonaute (Ago) proteins are present in all three domains of life (bacteria, archaea and eukaryotes) and use small (15–30 nucleotides) oligonucleotides as guides to bind complementary nucleic acid targets. According to their domain organization Agos are divided into long and short Agos. Long Agos are composed of 4 major functional domains (N – strand separation, PAZ (PIWI-Argonaute-Zwille)—guide strand 3′-end binding, MID (middle)—guide strand 5′-end binding, and PIWI (P element-induced wimpy testis)—nucleic acid cleavage, if it is active) and two structural linker domains L1 and L2. Long Agos include eukaryotic Argonautes (eAgos) that form the functional core of the RNA-interference (RNAi) machinery responsible for the regulation of gene expression, silencing of mobile genome elements, and defence against viruses. Based on their phylogeny, long pAgos can be subdivided into long-A and long-B containing and lacking catalytic DEDX tetrad in the PIWI domain, respectively, and play a role in host defense against invading DNA such as plasmids and viruses (1,2,3,4,5,6,7).

The majority (∼60%) of pAgos belong to the group of highly divergent short pAgos, which contain only the MID and inactive PIWI domains. In their operons or polypeptides, short pAgos are typically associated with APAZ (Analog of PAZ) domain-containing effector proteins that according to structure modelling and experimental structures correspond to the N, L1 and L2 domains of long pAgos (8,9,10,11,12). APAZ-containing proteins are often fused to effector Sir2 (Silent informator regulator 2), Mrr nucleases or TIR (Toll-Interleukin-1 Receptor) domains. Heterodimeric APAZ/Agos or single chain APAZ-Agos resemble long PAZ-less pAgos containing an additional effector domain. Upon guide-mediated detection of invading DNA by short pAgos, their associated effector enzymes kill the host cell and consequentially prevent the spread of the invader (9,13,14).

In this work, we focus on the truncated long-B (15) prokaryotic Argonaute AfAgo (also known as AfPIWI or Af1318 (16,17)) encoded by a hyperthermophilic archaeon *Archaeoglobus fulgidus* DSM4304 (15). Phylogenetically classified as a truncated long-B pAgo, AfAgo contains only MID and catalytically inactive PIWI domains in a single polypeptide chain, akin to typical short pAgos, and could therefore be considered a pseudo-short pAgo (8). As one of the first and one of the best structurally characterized prokaryotic Argonautes, AfAgo has been used as a structural model for studying Agos and Agos-nucleic acid interactions (16,17,18,19). However, the biological role of AfAgo remains elusive. The recently demonstrated AfAgo ability to form homodimeric assemblies that can bring together two copies of the guide-target duplex (20), and its intrinsic specificity to 3 nucleotides of both RNA guide and DNA target strands (21) distinguish it from previously characterized monomeric Argonaute proteins, that limit specific recognition of terminal nucleotides either to the guide strand (e.g. RsAgo, PDB ID: 6d8p (22)), or to the target strand (23), raising further questions regarding AfAgo functions *in vivo*.

Here, we restore the full open reading frame of the protein encoded upstream of AfAgo and confirm that it is the structural equivalent of the N-L1-L2 domains of long pAgo proteins. Furthermore, we use SAXS, X-ray crystallography, cryo-EM, and various biochemical and biophysical techniques to characterize the heterodimeric complex formed by AfAgo with the full-length upstream protein and compare its structural and functional properties to the previously characterized standalone AfAgo.

## Materials and methods

### Phylogenetic analysis

AfAgo close homologs were collected using a standard BLAST search against protein sequences in the NCBI Reference Sequence (RefSeq) database (https://www.ncbi.nlm.nih.gov/refseq/). Sequence redundancy was reduced by clustering homologs having 90% or more sequence identity with at least 90% alignment coverage using MMseqs2 (24). Cluster representatives were aligned using MAFFT (25) and those having only fragments within the MID-PIWI region were removed. Following this procedure, 200 sequences (AfAgo and its homologs) were selected for phylogenetic analysis. TtAgo (Long-A pAgo) homologs were collected in the same way and 20 sequences were selected. Joint MSA from both AfAgo and TtAgo homologs (220 sequences) was constructed using an accuracy-oriented MAFFT mode (L-INS-i) and only the MID-PIWI region of the aligned sequences was retained. The resulting MSA was processed with trimAl (26) to remove columns with excessive gap content followed by the phylogenetic tree inference using FastTree2 (27). The obtained pAgo tree was rooted at the midpoint and annotated using iToL v5 (28).

### Gene context analysis

To identify putative pAgo operons, the same proteins used for phylogenetic analysis were subjected to the gene neighborhood analysis using WebFlags (29). The analyzed neighborhood consisted of three genes upstream and three genes downstream of the pAgo gene. Proteins encoded by the upstream or downstream genes were further characterized based on their sequence and/or structure.

### Protein sequence and structure analyses

Sequence-based homology detection was performed using HHsearch (30). Potential transmembrane regions were predicted with DeepTMHMM (31). For structure-based analysis, AlphaFold models were either obtained from the EBI database (32,33) or constructed using the ColabFold implementation of AlphaFold (34). Structurally related proteins in PDB were identified by searches with Dali (35) and FoldSeek (36). Structures were analyzed with ChimeraX (37).

### Nucleic acids used

All synthetic DNA and RNA oligonucleotides used for cloning and EMSA were purchased from Metabion (Germany) and are listed in Supplementary Table S1.

### Protein and protein-nucleic acid complexes expression and purification

All plasmids used for protein expression were obtained using whole gene synthesis and cloning service provided by Twist Bioscience and are listed in Supplementary Table S2.

Proteins were expressed in *E. coli* strain BL21 (DE3). Cells were grown in LB broth in the presence of ampicillin at 37°C. Protein expression was induced with 0.2% L-arabinose, as appropriate for each vector, at cell culture *A*_600_ value of 0.5, cells were incubated for 4 h at 37°C and harvested by centrifugation.

*E. coli* cells expressing proteins were disrupted by sonication in buffer A (20 mM Tris–HCl (pH 8.0 at 25°C), 200 mM NaCl, 2 mM phenylmethylsulfonyl fluoride (PMSF), 5 mM 2-mercaptoethanol), heated for 30 min at 70°C and cell debris was removed by centrifugation. The collected supernatant was incubated for 1 h at 37°C with 1 mM EDTA (ethylenediaminetetraacetic acid) and RNase A/T1 (ThermoFisher Scientific) (1:100). Next, the protein solution was centrifuged. His_6_-AfAgo-N and His_6_-fAfAgo proteins were purified by chromatography through HisTrap HP chelating and HiTrap Heparin HP columns (Cytiva). scfAfAgo protein was purified by chromatography through HiTrap Heparin HP and HiLoad Superdex 200 columns (Cytiva). All purified proteins were of > 90% homogeneity as judged by SDS-PAGE. Proteins were stored at –20°C in a buffer containing 20 mM Tris–HCl (pH 8.0 at 25°C), 500 mM NaCl, 1 mM dithiothreitol (DTT) and 50% v/v glycerol. The identity of the purified proteins was confirmed by mass spectrometry. Protein concentrations were determined from A_280_ measurements using the theoretical extinction coefficients calculated with the ProtParam tool available at http://web.expasy.org/protparam/.

To obtain AfAgo-bound nucleic acids, *E. coli* BL21 (DE3) was transformed with pBAD_TwinStrep-fAfAgo, pBAD_TwinStrep-AfAgo or pBAD_TwinStrep-AfAgo + pCDFDuet_His-AfAgo-N plasmids. Cells were grown at 37°C in LB medium in the presence of ampicillin (pBAD constructs) or ampicillin and streptomycin (pBAD + pCDF constructs) until *A*_600_ value of 0.7 was reached. Then, expression was induced by adding 0.2% w/v l-arabinose with pBAD vectors or 0.5 mM IPTG and 0.2% l-arabinose with pBAD + pCDF vectors, and cells were harvested after 4 h. Cells were disrupted by incubating 1 h at 30°C in lysis buffer, containing 20 mM Tris–HCl (pH 8.0 at 25°C), 100 mM NaCl, 2 mM (PMSF), 5 mM 2-mercaptoethanol, 3 mg/ml lysozyme (ThermoFisher Scientific, cat#89833). The AfAgo-NA complex was purified using StrepTactin (pBAD constructs) or Histrap and StrepTactin (pBAD + pCDF constructs) columns, all buffer solutions contained 100 mM NaCl.

### Nucleic acid extraction and analysis

To extract nucleic acids co-purified with the AfAgo complexes, 1 ml of Roti-phenol/chloroform/isoamyl alcohol (Carl-Roth cat#A156) was added to the 1 ml of purified protein-NA fractions in 5PRIME Phase Lock Gel tubes (Quantabio cat#733-2477). The upper aqueous phase was isolated and 0.1 volumes of 1 M sodium acetate, 3 volumes of 100% ethanol and 10 μl glycogen (ThermoFisher cat#R0561) were added. This mixture was vortexed briefly and incubated at –20°C for 20 h. Samples were centrifuged for 20 min and the supernatant was removed from the pellet. The pellet was washed with cold (–20°C) 70% ethanol. The pellets containing the copurified nucleic acids were dried for 20 min at room temperature, and pellets were resuspended in 30 μl nuclease-free water.

Co-purified nucleic acids were dephosphorylated with FastAP Thermosensitive Alkaline Phosphatase (ThermoFisher cat# EF0651) and [γ-^32^P]-ATP (PerkinElmer) labelled with T4 polynucleotide kinase (PNK) (ThermoFisher cat#EK0031). Labelled nucleic acids were incubated with nucleases (ThermoFisher DNase I cat#18047019, RNase A/T1 cat# EN0551) for 60 min at 37°C. After nuclease treatment, samples were mixed with RNA Gel Loading Dye (ThermoFisher cat# R0641), heated for 5 min at 95°C and resolved on 20% denaturing (8 M urea) polyacrylamide gels. The molecular weight marker used for RNA size identification was Decade Marker System (Ambion cat#AM7778). Radioactivity was captured from gels using phosphor screens and imaged using a Typhoon FLA 7000 laser-scanner (GE Healthcare).

### RNA sequencing and analysis

RNA samples (without an additional PNK treatment) were converted to DNA libraries using a Small RNA-Seq Library Prep Kit (Lexogen cat#052). The concentration and quality of libraries were measured with Qubit Fluorometer (ThermoFisher) and 2100 Bioanalyzer (Agilent).

Libraries were sequenced using Illumina MiniSeq sequencing with single-end reads and 150 bp read length. Single-end reads were processed by trimming adapters with AdapterRemoval v2.3.043 (38). Then the processed reads were aligned to the *E. coli* strain K12 substrain BL21 (DE3) genome (NCBI: NZ_CP081489.1) and the additional pBAD_TwinStrep-fAfAgo, pBAD_TwinStrep-AfAgo, pCDFDuet_His-AfAgo-N plasmids (Supplementary Table S2) using BWA-MEM v0.7.1744 (39). In order not to filter out shorter reads during the alignment process, aligned reads with MAPQ values greater or equal to 15 were chosen. FastQC v0.11.845 (40) was used for read quality control and SAMtools v1.746 (41)—for indexing, sorting and analyzing alignment files. A custom script (fragmentation-bias.jl) (GitHub: https://github.com/agrybauskas/argonaute-bound-rna-manuscript) in combination with Weblogo v3.7.447 (42) were used to produce nucleotide frequency plots. Gene enrichment analysis was performed with bedtools v2.26.048 (43) and FPKM_count.py v4.0.0 of RSeqQC package (44).

### Mass photometry

Mass photometry measurements of fAfAgo were performed by Refeyn Ltd, using the ‘Refeyn OneMP’ system. Before measurement, protein stock solutions were diluted to 20 nM in a buffer, containing 20 mM Tris–HCl (pH 8.0 at 25°C), 500 mM NaCl.

### X-ray crystallography

AfAgo-N was concentrated to 9.2 mg/ml in a buffer containing 20 mM Tris–HCl (pH 8.0 at 25°C), 100 mM NaCl and 2 mM MgCl_2_. The fAfAgo complex was prepared by mixing AfAgo in the storage buffer supplemented with 5 mM MgCl_2_, with single-stranded DNA oligonucleotide GS-851 (Supplementary Table S3) followed by an equimolar amount of AfAgo-N protein. Glycerol was removed by passing through NAP Illustra columns (GE Healthcare) equilibrated with the ‘Low salt’ buffer (20 mM Tris–HCl (pH 7.5 at 25°C), 5 mM MgCl_2_, 150 mM NaCl, 2 mM DTT). The eluted complex was concentrated to 100 μM. Protein crystallization buffers are listed in Supplementary Table S3.

Datasets were collected at EMBL P13 and P14 beamlines at the PETRA III storage ring of the DESY synchrotron (Hamburg, Germany). Before cryo-cooling crystals were washed shortly in cryoprotection solution (Supplementary Table S3). All datasets were processed by XDS (45) followed by POINTLESS (46), AIMLESS and TRUNCATE (47). Phases for the fAfAgo complex with DNA were obtained by molecular replacement using MOLREP with AfAgo protein (PDB ID: 2bgg) as an initial model. Initial phasing yielded electron density for the AfAgo subunit, phosphorylated 5′-end of the DNA chain and magnesium ion bound at the C-terminus. After a few rounds of remodeling in COOT (48) and refinement by Phenix (49), the model was improved significantly, and part of the second DNA chain could also be modeled. This improved the phases further allowing to model a fragment of AfAgo-N protein. The resultant model was used as an initial model for phasing the P1 AfAgo-N dataset. The complete AfAgo-N structure was modeled by several rounds of passing the partial model between the two crystal structures, each step resulting in an improved model. Both AfAgo-N structures were refined by REFMAC (50).

The crystallization and cryoprotection solutions, dataset parameters, refinement statistics, and PDB accession codes are summarized in Supplementary Table S3.

### Small angle X-ray scattering of fAfAgo and scfAfAgo

SEC-SAXS data were collected at the P12 EMBL beamline of the PETRA III ring of DESY synchrotron (Hamburg Germany) equipped with a Pilatus6M (‘Dectris’) detector located at 3 m distance. The X-ray wavelength was 0.124 nm. The complex of AfAgo with 5′-phosphorylated oligoduplex MZ-1288 and AfAgo-N was prepared as described in ‘Crystallization’ section and concentrated to 59 μM. 100 μl of the sample was applied on the Superdex200 Increase 10/300 column (GE Healthcare) equilibrated with the ‘Low salt’ buffer. SEC run was performed using Wyatt-MALLS-DLS system (Agilent, Wyatt, (51)) directly connected to the P12 beamline. SAXS data were collected throughout the complete run (3000 frames, 0.995 s each). The gel filtration profile was analyzed by CHROMIXS software (52). Frames corresponding to the peak of the complex and background (58 and 117 frames respectively) were averaged with ATSAS v.2.8.4 software (53) and converted into absolute scale by PRIMUS v. 3.0.2 (r12592) (54).

Samples of similarly prepared scfAfAgo complexes with 14 bp (MZ-1288) and 11 bp (MZ-864) were concentrated stepwise to 1–3.7 mg/ml concentrations in the ‘Low salt’ buffer. Apo scfAfAgo sample was measured in the same buffer supplemented with 0.5 M NaCl. All samples were centrifuged at the maximal speed before data collection. The capillary of the automated sample changer (‘Arinax’) used in batch measurements was thermostated at 20°C. The SAXS data are presented in Supplementary Figure S2 A. 40 frames (0.05 s each) were collected from each sample and 80 frames of the corresponding buffer were processed by automatic beam line procedure (55) and converted into absolute scale (53).

All SAXS data were parametrized by GNOM v. 5.0 (r10552) (56) and the resultant curves were used for molecular mass estimations (ATSAS programs, (53)). *Ab initio* shape determination was performed with DAMMIF v. 3.0.2 (r12592) (57) and GASBOR v. 2.3i (r12592) (Figure 5B, C and Supplementary Figure S2). Experimental data were compared with protein chains of fAfAgo-DNA crystal structure using CRYSOL v. 2.8.3 (53), results are presented in Supplementary Figure S2.

### Cryo-EM data collection, image processing and model refinement

Two fAfAgo complexes with MZ-1800/MZ-1455 (g17/t17-bp) and MZ-1745/MZ-1747 (g30/t51-bp) guide-target heteroduplexes were mixed and concentrated to a concentration of 12 μM as described in SAXS sample preparation. The sample buffer contained 25 mM Tris–HCl (pH 8.5 at 25°C), 2 mM DTT, 5 mM MgCl_2_ and 125 mM KCl. Aliquots of 3 μl of each complex were applied on glow-discharged Copper R1.2/1.3 300 mesh holey carbon grid (Quantifoil) at 95% humidity and 4°C, after which samples were blotted and plunge-frozen in liquid ethane using Vitrobot Mark IV (Thermo Fisher Scientific, Waltham, USA).

Cryo-EM data of fAfAgo-NA complexes were collected with a Glacios microscope (Thermo Fisher Scientific, USA) operated at an accelerating voltage of 200 kV and images were collected using EPU 3 software (Thermo Fisher Scientific, USA) at a nominal magnification of 92 000× with a defocus in the range from –1 to –2 μm. The images were recorded on a Falcon III direct electron detector in the counting mode. A dose rate of 0.8 e^−^/px/s and an exposure time of 46.33 s were used. The recorded movies consisted of 30 frames with a total dose of ∼31 e^−^/Å^2^. A total of 2152 (fAfAgo-g30/t51) and 1140 (fAfAgo-g17/t17) movies were collected.

Cryo-EM data were processed using CryoSPARC v4.2.1 (58). The summary of image processing and 3D volume refinement is presented in Supplementary Figure S9, S10 and Supplementary Table S4. Particles in both datasets were blob-picked and extracted from dose-weighted and motion-corrected images. Several rounds of 2D classification were performed to remove junk particles.

Selected particles of fAfAgo-g30/t51 dataset were additionally sorted by heterogeneous refinement. The selected class contained 498 038 particles and was further refined by homogeneous refinement followed by local refinement within a solvent mask. The final reconstruction has a resolution of 2.81 Å at FSC GS threshold of 0.143. The best 2D classes of fAfAgo-g17/t17 dataset were finally sorted by *ab initio* reconstruction. The selected subset of 364005 particles was first used for homogeneous refinement and then for local refinement procedures. The final map has an FSC GS resolution of 3.56 Å.

Both cryo-EM maps were sharpened using phenix.auto_sharpen (59). The crystal structure of fAfAgo-DNA was used as an initial model (DNA chains removed) in both cases. Nucleic acid heteroduplexes were generated using Chimera v1.17.1 (60) and combined with the protein model in COOT (48). Models were refined using phenix.real_space_refine (61) and rebuilt in COOT.

### Electrophoretic mobility shift assay (EMSA)

Oligonucleotides used for EMSA (Supplementary Table S1) were 5′-labeled using [γ-^32^P] ATP (Perkin Elmer) and T4 polynucleotide kinase (ThermoFisher). Double-stranded substrates (Supplementary Table S1) were obtained by briefly heating a mixture of 5′-^32^P and 5′-P complementary oligonucleotides at a molar ratio of 1:1.2 to 95°C and annealing to room temperature over 4 hours.

EMSA reaction mixtures were prepared in dilution buffer (1 × TAE (40 mM Tris-acetate, 1 mM EDTA, pH 8.4 at 25°C, Invitrogen), 5 mM Mg (OAc)_2_, 0,1 mg/ml BSA, 1 mM DTT, 10% glycerol) and contained 0.01 nM of 5′-^32^P labeled oligonucleotide substrate and increasing concentrations of protein: 0, 0.05, 0.1, 0.2, 0.5, 1, 2, 5 nM for single-stranded substrates or 0, 0.1, 0.5, 1, 2, 5, 10, 20 nM for double-stranded substrates. To assess how binary pAgo:guide complex recognizes various targets, the binary complex was formed by mixing 400 nM of pAgo with 800 nM of 5′-P guide and incubating at room temperature (25°C) for 10 min. Binding reactions were prepared in dilution buffer and contained 0.01 nM of 5′-^32^P labeled target as well as increasing concentrations of the binary complex: 0, 0.005, 0.01, 0.02, 0.1, 0.5 nM. Control mixes were prepared by mixing either 0.5 nM of pAgo without the guide (C_P_) or 1 nM of guide (C_g_) with 0.01 nM of radiolabeled target. To assess the influence of a guide-target mismatch, a competition experiment was performed, where the competitor tDNAs had various mismatches in t1-t8 positions relative to the gRNA. To that end, the fAfAgo:guide complex was pre-formed as described above. Then, the 8 bp complementary 5′-^32^P labeled ssDNA target – the same used for guide-target experiments – was mixed with a competing 5′-P ssNA target. The fAfAgo:gRNA complex was then added to the mixture and incubated at room temperature for 10 min. The final reaction contained 0.1 nM 8 bp complementary 5′-^32^P labeled ssDNA target, 0.1 nM fAfAgo:gRNA complex, and varying concentrations of competitor tDNA: 0, 0.1, 0.5, 1, 5, 10, 50 nM. In all experiments, reaction mixtures were incubated at room temperature for 1 h prior to resolution on 8% polyacrylamide gels (29:1 acrylamide/bis-acrylamide in 1× TAE supplemented with 5 mM Mg (OAc)_2_). Electrophoresis was performed in electrophoresis buffer (1× TAE, 5 mM Mg (OAc)_2_). Radiolabeled substrates were detected by phosphor imaging using Amersham Typhoon laser-scanner. For competition experiments, densitometry was performed in OptiQuant 03.00 and K_I_ calculations performed in KyPlot 5.0. Data were analyzed as described previously (62).

### Atomic force microscopy experiments

Visualization of formed structures of fAfAgo protein complex and DNA fragment was performed using AFM as described previously (20). The reaction solution was prepared in HEPES buffer (33 mM HEPES, 66 mM KOAc, 5 mM Mg (OAc)_2_, pH 7.8 at 25°C) at a ratio 10:1 of protein complex:DNA substrate and incubated for 20 min at room temperature. Subsequently, glutaraldehyde was added as a crosslinker to a final concentration of 2.5%, promoting the protein–DNA binding through amide groups. Glutaraldehyde was quenched after 10 min with 10-fold dilution with Tris buffer (33 mM Tris-Acetate, 66 mM KOAc_2_, 5 mM Mg (OAc)_2_, pH 7.8). Afterwards, 10 μl of fAfAgo-DNA complex solution was spread out on APS-modified mica, which was prepared by incubating freshly cleaved mica sheets (IV grade, SPI Supplies. Inc., USA) in 0.17 mM APS solution (1-(3-aminopropyl)-silatrane 2,8,9-trioxa-5-aza-1-silabicyclo [3.3.3] undecane) for 30 min, to decorate the surface with –NH_2_ groups. After 2 min incubation, mica sheets with adsorbed DNA:protein complexes were rinsed with MilliQ water (18.2 MΩ × cm) and dried under nitrogen flow. Lastly, prepared mica sheets are visualized by an atomic force microscopy system DimensioIcon (Bruker, USA) in tapping mode in the air with probes of a nominal spring constant of 2–40 N/m. The image analysis was performed with WSxM (5.0) and NanoscopeAnalysis (1.9) software packages. The protein-DNA complexes were selected with no effect on DNA length. The theoretical length of 585 bp DNA is 195 nm. The recorded DNA length is 192.6 ± 5.9 nm (*n* = 89). Thus, only the structures that fulfilled this requirement underwent further analysis.

### Single-molecule FRET experiments

To probe the RNA-guided DNA targeting mechanism by (sc)fAfAgo, surface-immobilized molecule measurements with excitation in the total internal reflection (TIR) mode was performed on the same setup as described previously (20). Briefly, the objective used was 100 × 1.4 Oil Plan Apo VC (Nikon), excitation laser was reflected off the FF01-446/510/581/703, FF01-775/SP dichroic mirror (Semrock), the fluorescence signal was split by T640lpxr-UF2 dichroic mirror (Chroma), and the different spectral channels were projected on the same EMCCD (DU-897ECS0-UVB, Andor). Measurements of surface-immobilized DNA fragments were performed in a flow cell assembled from a six-channel Sticky-Slide VI 0.4 (Ibidi) and a coverslip functionalized with polyethylene glycol derivatives as described previously (63). The flow cell was incubated with 5 μg/ml of Neutravidin (Molecular probes) in TAE buffer (40 mM Tris-acetate, 1 mM EDTA, pH 8.4 at 25°C) for 2 min, washed with TAE, injected with 200 μl 10 pM pre-annealed biotinylated anchor ssDNA/target ssDNA duplex (MZ-1656/MZ-1715 for anchor/8 bp-complementary target and MZ-1656/MZ-1752 for anchor/non-complementary target) and flushed immediately with TAE. pAgo-guide complexes were formed by incubating a mixture of protein and MZ-1655 gRNA solutions in RB (TAE supplemented with 5 mM MgOAc_2_, 2.5 mM Trolox (Sigma-Aldrich), 1% w/v glucose (TCI Europe)) at 1 μM for 15 min at 25°C and were subsequently diluted in RB to a working concentration of 10 nM. Before measurement, a 10 nM solution of pAgo in RB supplemented with 15 U/ml glucose oxidase (Sigma-Aldrich) was injected into the cell. Trolox was treated with UV light for 20 min according to Cordes et al. (64).

Fluorescence burst data acquisition in alternating laser excitation (ALEX) mode for diffusing molecules to probe DNA looped states was based on Kapanidis et al. (65) and performed using the alternative functionality of the setup as described previously (20). Measurements were performed in a chambered coverglass well (155411, Nunc Lab-Tek, Thermo Scientific) using the same reaction buffer as above. The reaction volume was 200 μl. DNA concentrations were 17–50 pM. Measurements at different protein concentrations were carried out by adding small volumes of protein diluted in RB to the reaction. No oxygen-scavenging or triplet-quenching additives were used.

Single molecule data analysis was performed as described in detail previously (20). The donor and acceptor intensities were extracted for individual DNA fragments from each frame of the fluorescence movie. From the resulting intensity trajectories, we calculated the time courses of the proximity ratio, E. From the collection of E trajectories we calculated histograms of single-molecule population and time-averaged *E* values. To quantify the durations of interaction, we first idealized the E trajectories using HMM with a two-state model in QuB software, and then built the cumulative histograms of the interaction state durations.

## Results

### Restored ORF from *Archaeoglobus fulgidus* DSM4304 reveals a lost protein

**Figure 1.**
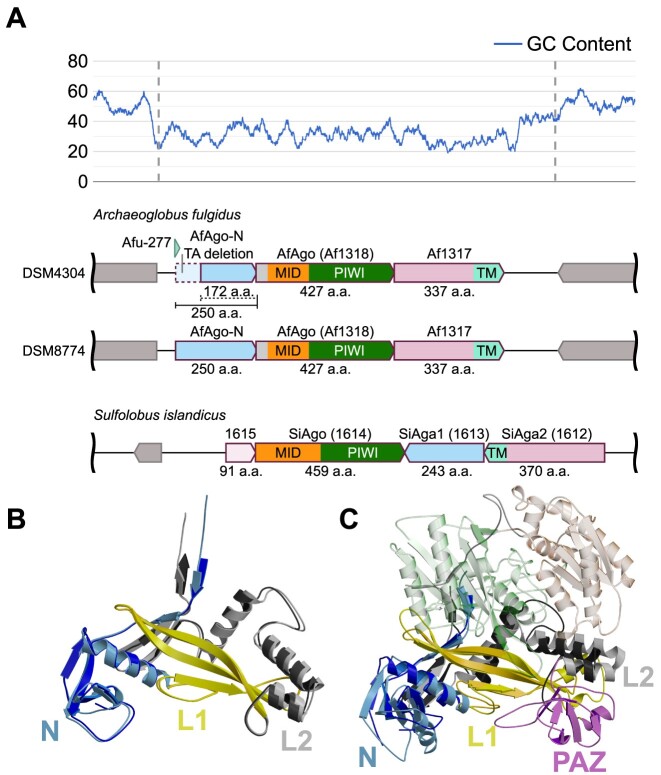
(**A**) Top: GC content of *Archaeoglobus fulgidus* DSM4304 genome region of interest, containing AfAgo and related genes. GC content values were smoothed using a simple running average over a 100 bp window. The dashed lines are a visual guide for the approximate boundaries of the lower-GC region. Bottom – genes located in the corresponding region of interest in *A. fulgidus* DSM4304 and DSM8774, along with a region of appropriate length from *Sulfolobus islandicus*, containing the SiAgo-Aga1-Aga2 system. (**B**) Comparison of AfAgo-N crystal structures. P1 structure is of darker shades. AfAgo-N and RsAgo proteins are colored according to the domain structure: N-terminal – blue, L1 linker – yellow, L2 linker – grey, MID – orange and PIWI – green. PAZ domain in RsAgo is colored pink. (**C**) AfAgo-N crystal structure (P1, 1.9 Å, darker colors) superimposed on the structure of RsAgo, a representative member of the long-B Ago family (PDB ID: 5awh, colored like in A). MID and PIWI domains of RsAgo are shown as transparent cartoons.

AfAgo (or Af1318)-encoding gene is located in a genetic island of the *Archaeoglobus fulgidus* DSM4304 genome with a lower GC content (∼31%) than the average GC content for the flanking genome sequences (∼50%) or the whole genome (∼53%, Figure 1A) (66), suggesting it was acquired by *A. fulgidus via* horizontal gene transfer (HGT) from an unknown low-GC content host. The AfAgo ORF overlaps with ORFs of two hypothetical proteins, suggesting that proteins encoded by all three operon-forming genes may act together (Figure 1A). The downstream 337 amino acid (aa) protein is similar (score 93.5, EMBOSS Needle) to the membrane effector SiAga2 of the antiviral SiAgo system from archaeon *Sulfolobus islandicus* (67). Conversely, the upstream ORF encodes a 172 aa putative protein that shows no similarities to proteins of known function. Comparison of this 172 aa ORF to a homologous sequence found in a nearly identical AfAgo-encoding genetic island present in the genome of a related strain *A. fulgidus* DSM88774 (Figure 1A) revealed that it contains a premature STOP codon due to a 2 bp deletion. The longer (250 aa) protein encoded by the *A. fulgidus* DSM88774 is similar (score 42.5, EMBOSS Needle) to SiAga1, a protein that forms a functional heterodimer with SiAgo (Figure 1A) (67). Notably, analysis of the small non-messenger RNA (snmRNA) from *A. fulgidus* DSM4304 identified an 80 nt snmRNA named Afu-277 transcribed from the upstream ORF region, confirming active transcription of the AfAgo operon under native conditions (68). Though Afu-277 was assigned to H/ACA small nucleolar RNAs, we propose here that it is merely a fragment of the upstream gene transcript: presumably, the premature STOP codon in *A. fulgidus* DSM4304 strain promotes degradation of the untranslated 3′ region of the upstream gene mRNA that remains unprotected from RNases (69). The above findings prompted us to test if the restored full-length upstream *A. fulgidus* DSM4304 protein forms a functional SiAgo/SiAga1-like heterodimeric complex with AfAgo (67).

### The AfAgo operon encodes an N-L1-L2 domain protein

To study the structure and function of the full-length upstream protein, the restored full-length open reading frame, with the missing TA nucleotides inserted, was cloned into an *E. coli* expression vector and the corrected protein was subsequently purified by liquid chromatography (Supplementary Figure S1). We were able to solve its structure by X-ray crystallography in two symmetry groups: P1 (resolution 1.85 Å) and P3_2_21 (resolution 1.4 Å) (see Materials and Methods and Supplementary Table S3 for details). Two full-length upstream protein chains found in the asymmetric unit of the P1 structure, and a single chain found in the asymmetric unit of the P3_2_21 structure overlap with an RMSD of 0.85 Å (determined using PDBe Fold (70), Figure 1B). The structurally closest proteins to the full-length upstream protein determined by DALI search (as a search model, a high-resolution structure P3_2_21 1.4 Å was used) are long-B RsAgo (PDB ID: 6d8p, *Z*-score 12.1) and long-A CbAgo (PDB ID: 6qzk, *Z*-score 10.1), confirming that it is a structural equivalent of N, L1 and L2 domains that are typical for the long pAgos. We henceforth refer to the full-length upstream protein as AfAgo-N.

### AfAgo and AfAgo-N form a heterodimer

To test if AfAgo and AfAgo-N form a functional complex, we engineered a pBAD expression vector where both AfAgo-N and AfAgo genes were placed under a single P_BAD_ promoter (Supplementary Table S2) and co-expressed both proteins in *E. coli*. AfAgo-N carrying an N-terminal His_6_-tag co-purified on the Ni^2+^-affinity column with AfAgo, confirming that AfAgo-N and AfAgo proteins form a stable complex (Supplementary Figure S1B-D). Mass photometry experiments indicated that the predominant population of AfAgo-N/AfAgo particles have a *M*_W_ that corresponds to the theoretical *M*_W_ of an AfAgo-N/AfAgo heterodimer (79.7 kDa) (henceforth – full AfAgo or fAfAgo) (Supplementary Figure S1A). Bioinformatic analysis (see below) shows that some AfAgo homologs are fused to their upstream AfAgo-N counterparts forming single polypeptides. To study a similar fAfAgo variant, in which AfAgo-N and AfAgo proteins are fused into a single polypeptide (henceforth – single-chain fAfAgo or scfAfAgo), the synthetic gene encoding scfAfAgo was expressed in *E. coli* and the protein was successfully purified (Supplementary Table S2, Supplementary Figure S1). SAXS measurements confirmed that fAfAgo heterodimer and scfAfAgo adopt a similar shape in solution (Supplementary Figure S2).

### fAfAgo uses an RNA guide to bind a DNA target

**Figure 2.**
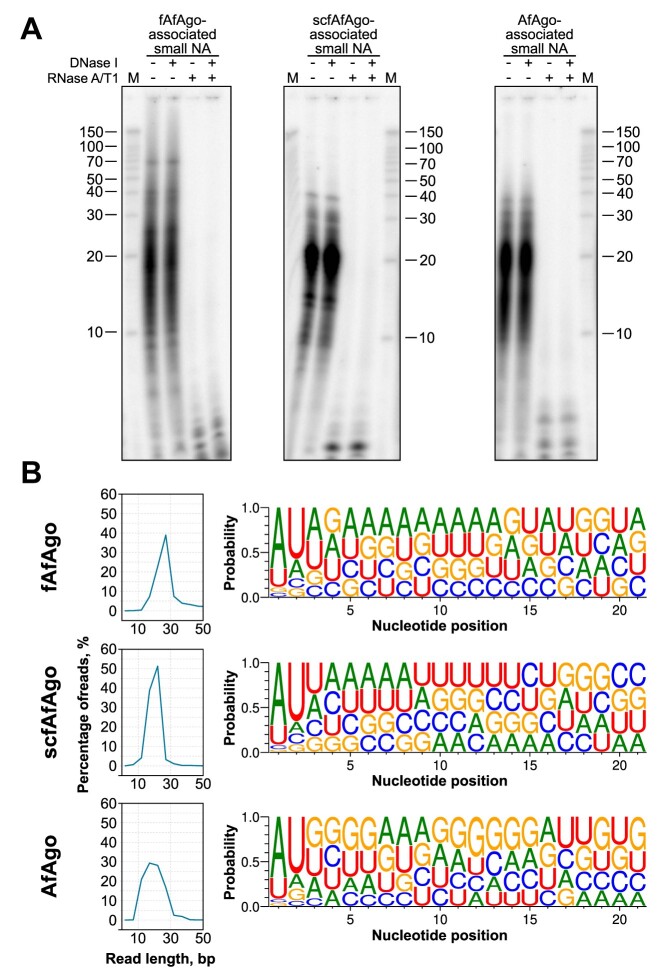
Nucleic acid binding by fAfAgo, scfAgo and AfAgo *in vivo*. (**A**) fAfAgo, scfAgo and AfAgo co-purify with small RNAs. Nucleic acids that co-purified with fAfAgo, scfAgo and AfAgo were [γ-^32^P]-ATP radiolabelled and treated with DNase I or RNase A/T1, or both, and resolved on a denaturing polyacrylamide gel. M, RNA ladder Decade Marker System (Ambion). (**B**) Length distribution (left) of small RNA co-purified with fAfAgo, scfAgo and AfAgo as determined by sequencing. Small RNAs associated with all AfAgo forms show 5′-AU preference (right).

Next, we characterized the nucleic acids bound to fAfAgo, scfAfAgo and AfAgo *in vivo*. To this end we extracted and sequenced NAs that co-purified with the respective AfAgo variants. Subsequent analysis revealed that all proteins associate with short RNA molecules (the predominant length being ∼20–30 for, fAfAgo and scfAfAgo, and ∼15–25 nt for AfAgo) that contain a 5′-phosphate (Figure 2A and B) and display a pronounced preference for the 5′-AU terminal dinucleotide (Figure 2B). These findings are in line with our previous study, where we demonstrated that AfAgo preferentially uses gRNA to recognize complementary tDNA (21).

Next, we characterized the nucleic acid binding properties of all three AfAgo variants *in vitro* by performing electrophoretic mobility shift assay (EMSA) experiments with single- and double-stranded substrates (Supplementary Figure S3). It is evident that both fAfAgo and scfAfAgo bind single-stranded (ss) nucleic acids with greater affinity than AfAgo. Binding affinity to ds substrates by all 3 proteins is less variable, but in all cases, we observe a notable preference for dsDNA and RNA/DNA heteroduplex substrates over dsRNA.

**Figure 3.**
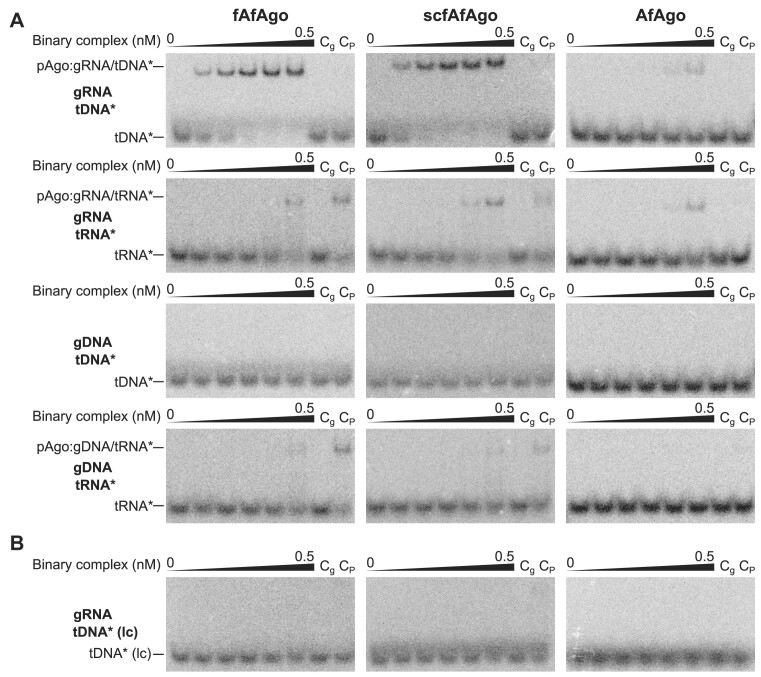
(**A**) Binding of 5′-^32^P labelled DNA and RNA targets by fAfAgo, scfAfAgo and AfAgo binary complexes preloaded with 5′-P RNA or DNA guides (molar AfAgo:guide ratio 1:2). Targets used are complementary to 1–8 positions of the guide strand. (**B**) Binding of 5′-^32^P labelled low complementarity DNA target by fAfAgo, scfAfAgo and AfAgo binary complexes preloaded with 5′-^32^P RNA or DNA guides (molar AfAgo:guide ratio 1:2). Low complementarity DNA target is only complementary to 4–7 positions of the guide strand. All experiments depicted were carried out in the same binary complex concentration range: 0, 0.005, 0.01, 0.02, 0.1, 0.5 nM. C_g_ control contains the highest guide concentration used (1 nM) and radiolabeled target, C_P_ control contains the highest protein concentration used (0.5 nM) and radiolabeled target.

To obtain a more accurate representation of nucleic acid binding by pAgos, which are usually first loaded with a single-stranded guide strand that is subsequently used to recognize the complementary target strand, we performed a variation of EMSA, where instead of apo-protein, we varied the concentration of pAgo preloaded with the guide strand (pAgo:guide binary complex). We find that for both fAfAgo and scfAfAgo the optimal combination is an RNA guide and a complementary DNA target since all other guide-target combinations resulted in suboptimal target binding (Figure 3A). In line with our previous results, AfAgo does not exhibit such clear selectivity, but its target binding is evidently weaker in comparison to fAfAgo and scfAfAgo (21). The affinity of all tested binary pAgo:gRNA complexes to DNA targets also depends on the guide:target sequence complementarity, as the low complementarity tDNA strands yielded no detectable ternary complexes (Figure 3B). Taken together, our EMSA studies suggest that fAfAgo and scfAfAgo are both more efficient at RNA-guided binding of complementary DNA targets than the previously studied standalone AfAgo (21).

To further investigate the impact of guide-target complementarity on target binding by AfAgo-guide complex, we performed a competition assay where the optimal ssDNA target was mixed with varying concentrations of competing target ssDNAs, bearing various mismatches in the critical seed region at tDNA positions t1 through t8 (Supplementary Figure S4). Results show that the guide-target mismatches have the highest impact at t2, t3 and t4 positions. Base t1 is likely less sensitive to a mismatch since it is bound in the specific binding pocket and is not involved in guide-target duplex formation. Interestingly, mismatches at positions t5-t8 have almost no impact on target binding affinity. Similar lack of sensitivity to t5–t8 mismatches is characteristic to some other pAgos, but not to eAgos (22,71,72,73,74).

**Figure 4.**
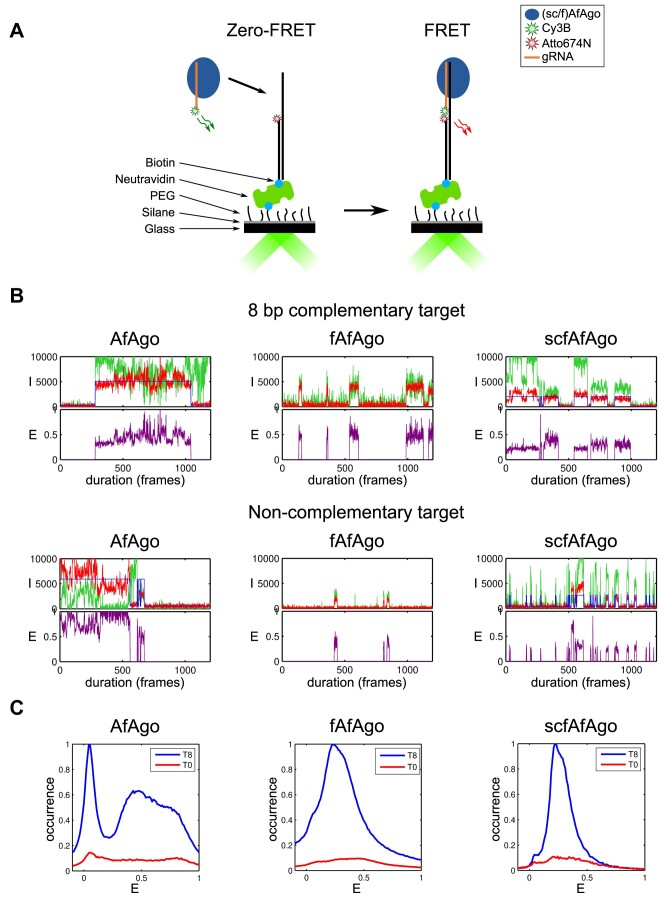
Single-molecule FRET studies of gRNA-loaded AfAgo, fAfAgo, and scfAfAgo interactions with surface-immobilized target DNA. (**A**) Schematic representation of the experimental setup. (**B**) Representative traces of donor (green) and acceptor (red) intensities and the corresponding proximity ratio E for 8 bp-complementary (top) and non-complementary (bottom) DNA targets. (**C**) Distributions of FRET efficiency for 8 bp-complementary (blue) and non-complementary (red) DNA targets. The distributions are scaled so that the ratio of their areas is equal to the ratio of the relative occurrences of high EFRET states.

To further investigate the RNA-guided DNA targeting mechanism of AfAgo, we utilized a single-molecule FRET assay. A surface-immobilized biotinylated DNA fragment (Figure 4A) was paired with a complementary RNA guide that had been pre-assembled with the AfAgo protein. We placed an acceptor fluorophore on the DNA and a donor fluorophore on the RNA at positions that would bring the two fluorophores into close proximity upon AfAgo-mediated RNA-DNA hybridization, facilitating efficient Förster resonance energy transfer (FRET) between them. We recorded fluorescence movies capturing the interactions between individual surface-immobilized DNA fragments and AfAgo-RNA complexes under various conditions, including different AfAgo variants and RNA-DNA complementarities (8 bp complementary and non-complementary targets). Figure 4B illustrates representative examples of the obtained FRET pair intensity trajectories along with the corresponding trajectories of the proximity ratio E. In these trajectories, we observe correlated and sudden changes in donor and acceptor intensities, indicating association and dissociation events between the AfAgo-RNA complex and the DNA fragment. When using non-complementary DNA, the periods of interaction are brief, suggesting weaker binding. Importantly, a control experiment involving DNA interaction with 8 bp complementary RNA guide alone did not yield trajectories with such coordinated changes in fluorophore intensity (Supplementary Table S5).

To evaluate the average values of the various E levels observed in individual trajectories, we have aggregated the selected E trajectories and calculated the resulting average within the initial 10 seconds (Figure 4C). Analysis of single-molecule populations and the time-averaged E values revealed prominent EFRET peaks at 0.5, 0.25, and 0.25 for AfAgo, fAfAgo, and scfAfAgo, respectively. For comparison, we also calculated E distributions for the DNA target with no complementarity to RNA. In these distributions, the high EFRET peak is noticeably less pronounced than in the corresponding distributions obtained with the 8 bp complementary DNA fragment, confirming that gRNA-tDNA complementarity is indeed required for the tDNA capture by the Argonaute-gRNA complex. Different EFRET peak positions observed for AfAgo (0.5) and (sc)fAfAgo (0.25) also suggest that the overall conformation of the gRNA-tDNA heteroduplex may differ in the complexes with the standalone AfAgo and the AfAgo-N-containing fAfAgo complexes.

### fAfAgo heterodimer structurally resembles long-B PAZ-less pAgos

Next, we obtained cryo-EM structures of the fAfAgo complex with two variants of gRNA/tDNA heteroduplexes: g30/t51 (30 nt gRNA, 51 nt tDNA, final resolution 2.83 Å) and g17/t17 (17 nt gRNA, 17 nt gDNA, resolution 3.56 Å), and a 2.5 Å crystal structure of fAfAgo bound to a 15 nt 5′-phosphorylated ssDNA oligonucleotide (see Materials and methods, Supplementary Figure S9, Supplementary Figure S10, and Supplementary Table S4). The nucleic acids resolved in the cryo-EM maps include a 25 bp protein-interacting fragment of gRNA/tDNA heteroduplex in the g30/t51 structure, and the full 17 bp gRNA/tDNA heteroduplex in the g17/t17 structure. fAfAgo proteins in these structures overlay with an RMSD value of ∼1.2 Å. The crystal structure contains fragments of two partially annealed non-self-complementary DNA oligonucleotide strands forming a short (3 bp) guide/target-like dsDNA region. Relative orientation of AfAgo and AfAgo-N proteins in the crystal structure slightly differs from that in cryo-EM structures obtained with longer gRNA/tDNA heteroduplexes (RMSD values 1.9–2.3 Å) (Supplementary Figure S12).

**Figure 5.**
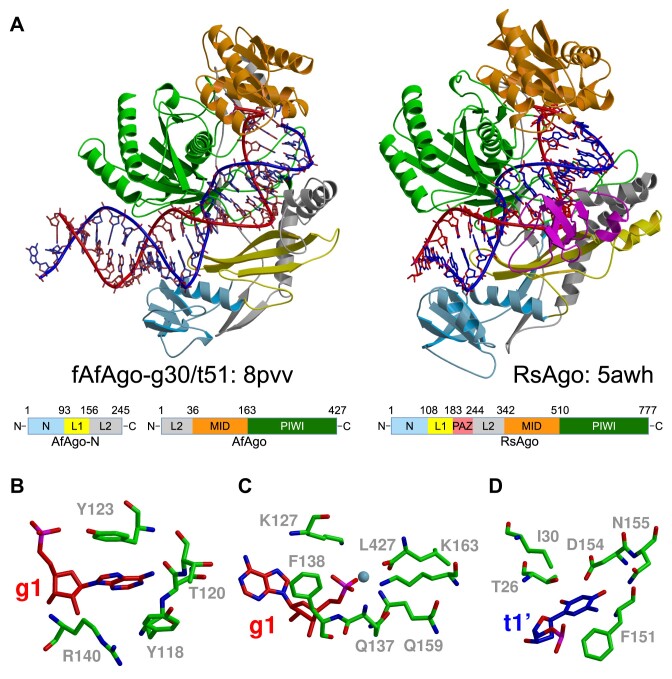
Cryo-EM structure fAfAgo-g30/t51. (**A**) Top – fAfAgo-g30/t51 complex (left, PDB ID: 8pvv) compared to RsAgo (right, PDB ID: 5awh). ‘Guide’ RNA strands are colored red, ‘target’ DNA strands – blue. Bottom – domain architecture of AfAgo-N and AfAgo. Domain architecture of RsAgo (PDB ID: 5awh) is shown for comparison. Proteins are colored according to the domain scheme. (**B**) The binding of 5′-phosphate of the guide strand. (**C**) Residues forming the first guide nucleotide binding pocket. (**D**) The binding of the first target nucleotide in the "side pocket’.

We will base our further discussion of the fAfAgo structures on the higher resolution cryo-EM structure with the longer (g30/t51) gRNA/tDNA. The contact surface area between AfAgo and N-AfAgo proteins in this complex, as calculated by the PISA server (75), is 1380 Å^2^, and includes 15 H-bonds, indicative of stable association. Interestingly, the C terminus of AfAgo-N in the fAfAgo structure is adjacent to the N-terminus of Ago subunit (Supplementary Figure S6B), implying that both proteins could have evolved from a single polypeptide, and also accounting for the fully functional scfAfAgo fusion protein (Figures 2–4). The AfAgo-N subunit sterically masks the AfAgo surface that was previously implicated in AfAgo homodimerization (20), making AfAgo oligomerization across this surface unlikely. This is in line with our atomic force microscopy (AFM) and single-molecule Förster resonance energy transfer (smFRET) data, which show that unlike the previously studied standalone AfAgo, fAfAgo and scfAfAgo do not form looped complexes with dsDNA (Supplementary Figure S7, S5). The overall structure of fAfAgo heterodimer aligns well with the structure of long-B Argonaute RsAgo (Figure 5A). As predicted, AfAgo superimposes with MID and PIWI domains of RsAgo, whereas AfAgo-N superimposes with the N-terminal domain and the L1-L2 linker domains. Only the RsAgo PAZ domain has no structural counterpart in the fAfAgo complex (Figure 1C, Figure 5A) (17).

Similarly to RsAgo, fAfAgo binds the gRNA/tDNA heteroduplex in the positively charged cleft, which is formed by both AfAgo and AfAgo-N subunits, forming multiple van der Waals and H-bond interactions to the RNA/DNA backbone (Figure 5, Supplementary Figure S9, S10). Such NA duplex binding greatly differs from the overall ds NA position in the crystal structures obtained previously with an isolated AfAgo protein, e.g. PDB ID: 6t5t and 6tuo (16,17,18,19,21) (Supplementary Figure S11A), where only the 5′-end of the guide/target duplex contacts AfAgo, the remaining part pointing away from the protein. As the fully formed fAfAgo NA-binding cleft may protect longer RNAs from cellular ribonucleases, this may also explain why fAfAgo and scfAfAgo co-purify from *E. coli* bound to longer RNAs (∼20–30 nt) than the stand-alone AfAgo (∼15–25 nt) (Figure 2).

Since the 5′-terminal gRNA nucleotide (adenosine) in the cryo-EM fAfAgo structures reported here is equivalent to gDNA nucleotide (deoxyadenosine) in the recently solved crystal structures of the standalone AfAgo bound to dsDNA (PDB ID: 6xu0 and 6xup (21)), we observe similar contacts of the protein to the 5′-terminal part of guide and the complementary target strands. The AfAgo subunit, both standalone and AfAgo-N-bound, anchors the 5′–phosphorylated terminus of the guide strand in a conserved MID domain pocket, disrupts the g1:t1 (the first guide strand and the complementary target strand nucleotides) base pair by placing the g1 adenine and the t1 thymine bases into separated protein pockets, and makes similar contacts to the neighboring g2:t2 and g3:t3 base pairs (Supplementary Figure S8A). A similar set of contacts to bases and backbone is also observed in the crystal structure of fAfAgo bound to a short DNA duplex fragment (Supplementary Figure S8).

Taken together, structures presented here demonstrate that fAfAgo heterodimer preserves the g1:t1 base pair recognition mechanism previously demonstrated for the standalone AfAgo, but unlike the isolated AfAgo subunit, fAfAgo binds the guide/target heteroduplex in a specialized cleft in a similar manner as the structurally related long-B pAgos.

### AfAgo homologs include both split and single-chain pAgos with various degrees of PAZ reduction

To better understand the relationship between AfAgo and other long-B pAgos, we collected a non-redundant set of AfAgo homologs that have associated genomic DNA sequences. We reasoned that other so-called ‘truncated’ long-B pAgos, similarly to AfAgo, may also have an upstream gene encoding for the missing N-terminal part. We also aimed to explore whether long-B pAgos are part of putative operons similar to AfAgo, which is associated not only with the upstream N-AfAgo, but also with a downstream gene (Af1317), encoding an uncharacterized protein.

**Figure 6.**
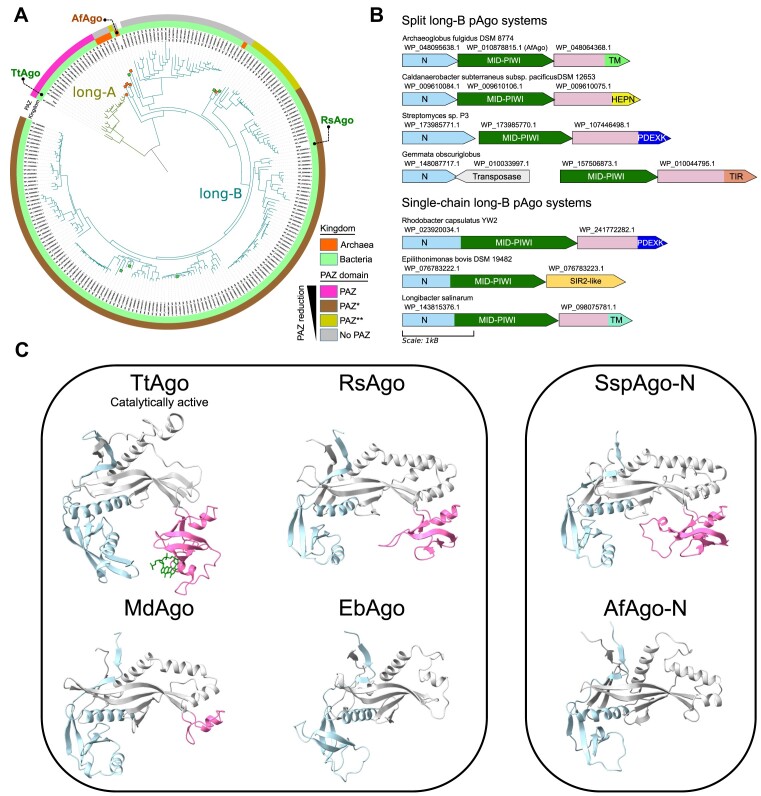
(**A**) Phylogenetic tree of 220 pAgo proteins constructed with Fasttree and rooted arbitrarily (midpoint root). Colored squares on branch leaves represent split pAgos (N and MID-PIWI regions are separate proteins). Branches for long-A and long-B pAgos in the tree are shown by different colors. Archaeal and bacterial proteins are indicated on the inner circle using orange and green colors respectively. The presence and the type of the PAZ domain is indicated on the outer circle: magenta, classical PAZ as in long-A TtAgo; brown, incomplete PAZ domain (PAZ*) as in RsAgo; yellow, remnants of PAZ domain (PAZ**); gray, PAZ domain is absent. (**B**) Composition of representative split and single-chain pAgo systems taken from the MID-PIWI phylogenetic tree. Colored tags represent genes coding for N-domains of split pAgos (light blue), MID-PIWI (dark green) and putative effectors (a mixture of pink and other colors). Putative effectors have a common alpha-helical region (pink) followed by effector domain. TM, putative transmembrane domain; HEPN, nuclease domain; PDEXK, PD- (D/E)XK nuclease domain, TIR, TIR domain, SIR2-like, Sir2 family protein. Protein IDs are indicated above each gene. (**C**) Examples of the PAZ domain present/absent in the structures of both single-chain and split pAgos. PAZ domain or its remnant are shown in magenta, N-domain is colored light blue, L1 and L2 regions are colored gray. In the case of single-chain pAgos, the MID-PIWI region is removed for clarity. Left – single-chain long-A pAgo: TtAgo, Thermus thermophilus Ago (PDB ID: 3dlh); single-chain long-B pAgos: RsAgo, *Rhodobacter sphaeroides* Ago (PDB ID: 5awh); MdAgo, *Maribacter dokdonensis* Ago (WP_074671526.1, AF structural model AF-A0A1H4M537-F1); EbAgo, *Epilithonimonas bovis* Ago (WP_076783222.1, AF structural model AF-A0A1U7PYU4-F1). Right – split long-B pAgos: SspAgo-N, *Streptomyces* sp. P3 Ago-N (WP_173985771.1) structure derived from the full Ago heterodimeric AF model (WP_173985771.1 + WP_173985770.1); AfAgo-N, *Archaeoglobus fulgidus* Ago-N subunit (structure determined in this study).

First, we constructed a phylogenetic tree that includes closely related AfAgo homologs (long-B pAgos) and a small set of close TtAgo homologs representing long-A pAgos (Figure 6A). We found that ‘truncated’ long-B pAgos are present in both archaea and bacteria and are not confined to a single clade. Next, we analyzed the genome neighborhood and found that similarly to AfAgo these ‘truncated’ pAgos upstream have a gene encoding the N-terminal part of a full-length long-B pAgo (Figure 6B). Thus, it appears that at least some of the ‘truncated’ long-B pAgos represent split pAgo systems and that this splitting occurred multiple times independently. To further substantiate this finding, we constructed AlphaFold structural models for some of the split pAgo representatives and found that, similarly to the full AfAgo, they form heterodimeric structures.

Previously, it was observed that long-A pAgos (e.g. TtAgo) have a canonical PAZ domain, whereas long-B pAgos such as RsAgo have a reduced PAZ (often annotated as PAZ*), which lacks structural elements for binding the 3′-end of the guide strand (4). On the other hand, AfAgo-N characterized here lacks the PAZ domain altogether. To investigate whether the presence of a PAZ domain depends on the long-B pAgo type (split or a single-chain), we have explored the corresponding regions using sequence alignments and structural models. Interestingly, we found that both single-chain and split long-B pAgos may either have or lack the PAZ* domain (Figure 6C). We also identified instances where the PAZ* domain has undergone further reduction, leaving only a single α-helix (Figure 6C). Taken together, these observations indicate that long-B pAgos, regardless of whether they are split or not, may have experienced various levels of PAZ degradation, including its complete loss.

Gene neighborhood analysis additionally revealed a conserved association of long-B pAgos (both split and single-chain) with a protein-coding gene located immediately downstream (Figure 6B). We found two major types of these associated proteins. The first type, represented by Af1317, corresponds to a fusion of a conserved α-helical domain with an additional functional domain such as putative transmembrane (TM), TIR, HEPN or PD- (D/E)XK domain. The second type corresponds to a Sir2-like protein similar to the ThsA protein from the Thoeris antiphage defense system (76). This observation suggests that catalytically inactive long-B pAgos may function by regulating activities of these effectors that upon activation may act as toxins.

## Discussion

### AfAgo – not so different after all?

As one of the first and the best structurally characterized Ago proteins, AfAgo has long been used as a structural model to study Agos and Agos-NA interactions (4,16,17,18,19,77,78,79,80,81). However, AfAgo differs significantly from most other pAgos. First, AfAgo consists only of MID and inactive PIWI domains. AfAgo thus resembles typical short pAgos, but it is phylogenetically closer to long-B pAgos and is therefore classified as a truncated long-B pAgo. Second, unlike other long pAgos that function as monomers, AfAgo is a dimer capable of binding two ends of a DNA fragment to form a looped complex (20). Third, unlike other structurally characterized pAgos that recognize only the terminal nucleotide of the guide and/or target, AfAgo specifically interacts with three nucleotides in both the guide and target strands (21). Finally, structural analysis of AfAgo complexes with NA shows that only the ds terminus of the guide-target duplex makes contacts with the MID domain, while its remaining part points away from the protein. In this aspect, AfAgo differs from other pAgos-NA complexes, where the guide-target duplex is tightly bound in the protein's NA interaction groove.

We show here that the unique AfAgo properties listed above by and large derive from the fact that previous studies treated AfAgo as an isolated standalone protein, neglecting hypothetical proteins encoded in the same operon. Indeed, we demonstrate here that AfAgo forms a heterodimeric complex with a reconstituted protein encoded upstream of AfAgo in the same operon of *A. fulgidus* DSM4304 strain. The upstream protein (AfAgo-N) is the structural equivalent of the N-L1-L2 domains of long pAgo proteins. In this way, the AfAgo-N/AfAgo heterodimeric complex (fAfAgo) structurally resembles a long PAZ-less pAgo. fAfAgo is most similar to the long-B RsAgo, in which the PAZ domain is smaller than in other long-A pAgos (15). Full AfAgo, like other long pAgos, forms a deep groove for interaction with the guide-target duplex that is absent in the standalone AfAgo. Additional protein-NA interactions provided by the fAfAgo groove may contribute to enhanced guide and target binding as the fAfAgo-gRNA complex forms much tighter complexes with target DNA than the standalone AfAgo (Figure 3, (21)). Nevertheless, fAfAgo, like the standalone AfAgo protein, requires a 5′-terminal gRNA adenine (gA1) (Figure 2B). The functional significance of such preference remains unclear. A possible explanation is that fAfAgo has adapted to use gRNAs that are generated *in vivo* by RNase E, which preferentially cleaves RNA at A/U rich sequences (82).

In the fAfAgo structure, AfAgo-N interacts with the AfAgo surface through which standalone AfAgo forms a homodimer, preventing AfAgo homodimerization. However, it remains an open question if in the native hyperthermophilic host *A. fulgidus* there is a dynamic equilibrium between fAfAgo heterodimer and AfAgo homodimer depending on cellular and environmental conditions, and if both AfAgo homodimer and fAfAgo heterodimer have distinct roles *in vivo*.

Structurally, fAfAgo heterodimer is also similar to *bona fide* short pAgos (Supplementary Figure S6, (9)). Since in both fAfAgo and short pAgo heterodimeric complexes the C-termini of the N-L1-L2 domain proteins are located close to the N-termini of the Ago proteins (Supplementary Figure S6), and some short pAgos even form single functional polypeptides together with their upstream encoded APAZ (N-L1-L2)-effector proteins, it is likely that single polypeptide pAgos were split into two proteins over the course of evolution. Apparently, such splitting occurred independently among long-B pAgos (exemplified by the AfAgo system studied here) and short pAgos (e.g. JomAgo, PgAgo (8)). To compare the native split fAfAgo variant to its hypothetical single-chain predecessor, we have constructed scfAfAgo, in which the AfAgo-N and AfAgo proteins are fused into a single polypeptide. In all our assays, scfAfAgo behaved similarly to fAfAgo (Figure 3, Supplementary Figure S3), providing no explanation for the potential benefits of the native split variant. Given the co-existence of both single-chain and heterodimeric short pAgos, one may assume that there is no significant functional difference between the active proteins composed of one or two polypeptide chains.

### Evolutionary origins of long-B, split long-B and short pAgos

**Figure 7.**
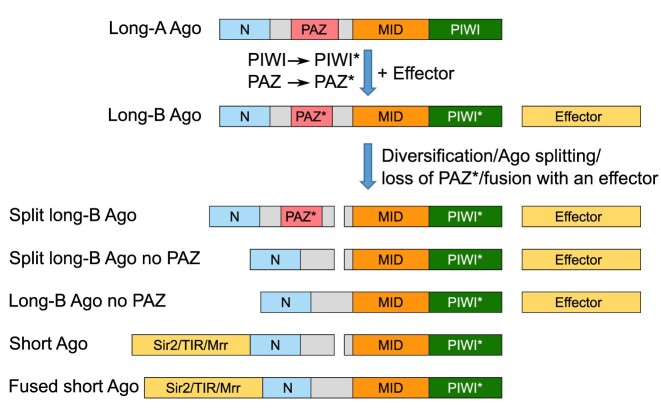
Proposed scenario for the emergence of diverse prokaryotic Argonautes. Following mutations in the PIWI domain and truncation of PAZ, long-A pAgo becomes catalytically inactive, but still able to bind the nucleic acid template (long-B pAgo). Long-B pAgos underwent diversification in several ways: (i) splitting pAgo into two proteins, N-Ago and Mid-PIWI-Ago, (ii) reduction or complete loss of the PAZ* domain, (iii) association with a functional effector either as a separate protein or as a fusion with the N-terminal region of Ago.

Our structural and functional characterization of the restored full AfAgo complex coupled with computational analysis of its homologs indicates that long-B Agos can be divided into two major groups: typical single-chain pAgos exemplified by RsAgo, and split pAgos, exemplified by full AfAgo. Full AfAgo and other split long-B Agos are similar to short pAgos, which also assemble into functional heterodimeric complexes composed of an APAZ-containing protein (a structural equivalent of the N-L1-L2 domains (9,10,11,12)) and a MID-PIWI protein. The major difference between the short and fAfAgo-like split pAgo systems is that the N1-L1-L2 subunit in short pAgo systems is often fused to an effector domain. However, we show here that fAfAgo and its homologs also have putative effector proteins encoded downstream of Ago, and thus may form functional complexes. Other common features shared by long-B Agos and short pAgos are the catalytically inactive PIWI domain and the lack of the canonical PAZ domain. The N1-L1-L2 subunit in short pAgos studied to date lacks the PAZ domain entirely as does full AfAgo. Other long-B Agos are also either PAZ-less or have a reduced PAZ domain lacking the pocket for binding the 3′-end of the guide strand. Based on these observations, we propose a likely scenario for the emergence of both catalytically inactive long-B and short pAgos from the catalytically active long-A pAgos (Figure 7, Supplementary Table S6). In this scenario, following the inactivation of the PIWI domain, the PAZ binding pocket for the 3′-end of the guide strand, which in catalytically active Agos limits the guide strand length, thereby enabling dissociation of target strand cleavage products, is no longer needed and is either reduced or lost altogether. As the PIWI domain became inactive, pAgos acquired new functionality *via* association with other effectors, either as separate proteins encoded in the same operon, or *via* a fusion to the pAgo N-terminus. Splitting of pAgos into two halves (the N1-L1-L2 and the MID-PIWI subunits) apparently occurred multiple times independently as both split and single-chain forms are present in long-B and short pAgos. The proposed scenario explains the observed diversity of short and long-B pAgos and the repurposing of these pAgos as regulators of toxic effectors that are unleashed by invading foreign nucleic acids.

### Full AfAgo-like systems in antiviral defense

The repaired AfAgo operon from *A. fulgidus* DSM4304 is analogous to the previously characterized antiviral system SiAgo from *S. islandicus* (apart from the putative regulator) (Figure 1A). The AfAgo-N protein is similar to SiAga1, which forms a heterodimeric complex with SiAgo. Therefore, the AfAgo-N/AfAgo heterodimer can be regarded as a structural equivalent of the SiAgo/SiAga1 complex (Supplementary Figure S6C). We hope that further structural and functional studies of AfAgo operon proteins, in particular, the association of fAfAgo heterodimer with the Af1317 protein (a homolog of the membrane effector SiAga1) encoded downstream AfAgo in the same operon, will shed light on the potential function and mechanism of the AfAgo system.

## Supplementary Material

gkad1241_Supplemental_File

## Data Availability

Cited: *Archaeoglobus fulgidus* DSM 4304 genomic sequence, GenBank accession no. NC_000917.1. strain Archaeoglobus fulgidus DSM8774 www.ncbi.nlm.nih.gov/nuccore/NZ_CP006577.1 *Sulfolobus islandicus* M.16.4 NC_012726.1 SiAgo ACR42215.1 SiAga1 ACR42214.1 SiAga2 ACR42213.1 AfAgo (Af1318) WP_010878815.1 Af1317 WP_048064368.1 AfAgo-N truncated WP_048064370.1 AfAgo complexed with a 16 nt DNA duplex, PDB ID: **2w42**. AfAgo complexed with 21 nt RNA duplex, PDB ID: **1ytu**. Ternary RsAgo complex containing guide RNA paired with target DNA, PDB ID: **6d8p** and **5awh**. Obtained in this work: SAXS data of fAfAgo heterodimer with 14 bp DNA oligoduplex (MZ-1288), SASBDB ID: *SASDRX8*. SAXS data of apo scfAfAgo, SASBDB ID: *SASDR29*. SAXS data of scfAfAgo complex with 11 bp DNA (MZ-864), SASBDB ID: *SASDRZ8*. SAXS data of scfAfAgo complex with 14 bp DNA (MZ-1288), SASBDB ID: *SASDRY8*. X-ray structure of AfAgo-N, PDB IDs: **8old (**P1, 1.9 Å) and **8olj (**P 3_2_ 2 1, 1.4 Å). X-ray structure of fAfAgo heterodimer with DNA, PDB ID: **8ok9** (P 2_1_ 2_1_ 2_1_). Cryo-EM structure of fAfAgo heterodimer with RNA/DNA heteroduplex, PDB ID: **8pvv** / EMDB ID: **EMD-17973** (2.8 Å) and PDB ID: **8qg0** / EMDB ID: **EMD-18386** (3.54 Å). Co-purified small RNA sequencing data are available on the NCBI Sequence Read Archive, under BioProject ID: PRJNA978552, https://www.ncbi.nlm.nih.gov/bioproject/PRJNA978552. Further data underlying this article is available in Zenodo at https://doi.org/10.5281/zenodo.10371518.
