# Supplementary material for: The missing part: the Archaeoglobus fulgidus Argonaute forms a functional heterodimer with an N-L1-L2 domain protein
Source: Nucleic Acids Res. 2024 Jan 9;52(5):2530–45. doi: 10.1093/nar/gkad1241 (PMC10954474; doi:10.1093/nar/gkad1241)
Supplement: gkad1241_Supplemental_File [file gkad1241_supplemental_file.pdf]

The missing part: the *Archaeoglobus fulgidus* Argonaute forms a functional heterodimer with an N-L1-L2 domain protein

Elena Manakova[1#], Edvardas Golovinas[1#], Reda Pocevičiūtė[1#], Giedrius Sasnauskas[1], Arunas Silanskas[1], Danielis Rutkauskas[1,2], Marija Jankunec[1,3], Evelina Zagorskaitė[1], Edvinas Jurgelaitis[1], Algirdas Grybauskas[1], Česlovas Venclovas[1], Mindaugas Zaremba[1*]

[1]Institute of Biotechnology, Life Sciences Center, Vilnius University, Sauletekio av. 7, LT-10257, Vilnius, Lithuania.

[2]Institute of Physics, Center for Physical Sciences and Technology, Savanoriu 231, LT-02300, Vilnius, Lithuania.

[3]Institute of Biochemistry, Life Sciences Center, Vilnius University, Sauletekio av. 7, LT-10257, Vilnius, Lithuania.

*To whom correspondence should be addressed. Tel: +370-5-2234357; Fax: +370-5-2234367; Email: mindaugas.zaremba@bti.vu.lt.

[#]These authors contributed equally: Elena Manakova, Edvardas Golovinas, Reda Pocevičiūtė.

SUPPLEMENTARY INFORMATION

Atomic force microscopy measurements

The fAfAgo protein complexes were adsorbed on freshly cleaved mica and visualized by AFM to determine the morphology and height distribution. Supplementary Figure S7A and B shows that the initial heterodimeric protein solution is heterogeneous with structures of 1-3 nm in height. Thus, we would expect that the same heterogeneity would be demonstrated for protein-DNA

structures. These products of protein-DNA binding are represented in Supplementary Figure S7C. We observed protein complexes sitting in the random spots within the whole DNA length, the majority at one or two ends of the substrate and only a few in the closed loop shape. AFM is a surface-sensitive technique, but only well-adsorbed molecules could be visualized. The crosslinker is used to fix unstable structures during AFM preparation.

When the effect of glutaraldehyde was quenched, we observed a severe decrease in the number of complexes, which prolonged the data collection by AFM, while not quenching the glutaraldehyde resulted in a surface more densely covered in fAfAgo:DNA complexes (Supplementary Figure S7C). However, we observed only negligible differences in the complexes formed. Previously studied homodimer (WT AfAgo) forms loop-shaped structures upon binding with DNA (50.8%), binds one of the DNA ends (35.3%), and both ends (13.1%). In this work, fAfAgo prepared by quenching the crosslinker, forms looped structures in 5.7% of cases, is bound to one of DNA ends (77.1%) and to both ends (17.1%). Similar results were observed when the crosslinker was not quenched: attached to the one end 72.2%, randomly 24.1%, and 3.7% as a loop. These results show that, as expected, fAfAgo lost the ability to form loop-shaped structures. Thus, 75% of total observed complexes were with protein complex bound to the DNA end; 20% of the population had protein the complex bound randomly or at both DNA ends, and only 5% were identified as forming a loop.

Supplementary tables

Supplementary Table S1. Oligonucleotides used in this work.

Oligonucleotide	Sequence (5′→3′ direction)	Description
Oligonucleotides used for crystallization and SAXS experiments		
GS-851	ATCGACCAGGCTACG	15 nt 5′-phosphorylated DNA oligonucleotide used for crystallization experiments of fAfAgo.
MZ-864/865	5′P-ATTATAATAGG<br>3′-TAATATTATCC	11 bp 5′-phosphorylated DNA duplex used for SAXS
MZ-1288	ATTGTGGCCACAAT	14 nt 5′-phosphorylated DNA oligonucleotide used for SAXS experiments.
Oligonucleotides used for EMSA experiments		

ssRNA (MZ-1480)	AUUGUACACGGCCGAAU	17 nt RNA oligonucleotide used as ssRNA and gRNA
ssDNA (MZ-1447)	ATTGTACACGGCCGAAT	17 nt DNA oligonucleotide used as ssDNA and gDNA
tDNA (MZ-1560)	CGGAATATATGTACAAT	17 nt DNA oligonucleotide used as tDNA, complementary to g1-g8 bases
tRNA (MZ-1556)	CGGAAUAUAUGUACAAU	17 nt RNA oligonucleotide used as tRNA, complementary to g1-g8 bases
tDNA (lc)<br>(MZ-1561)	CGGAATATTGGTACCCG	17 nt DNA oligonucleotide, used as low complementarity tDNA, complementary to g4-g7 bases
MZ-1481	AUUCGGCCGUGUACAAU	17 nt RNA oligonucleotide complementary to MZ-1480 and MZ-1447
MZ-1455	ATTCGGCCGTGTACAAT	17 nt DNA oligonucleotide complementary to MZ-1480, MZ-1447, and MZ-1800.
dsRNA	5′-AUUGUACACGGCCGAAU-3′<br>3′-UAACAUGUGCCGGCUUA-5′	MZ-1480 and MZ-1481 annealed
dsDNA	5′-ATTGTACACGGCCGAAT-3′<br>3′-TAACATGTGCCGGCTTA-5′	MZ-1447 and MZ-1455 annealed
RNA/DNA	5′-AUUGUACACGGCCGAAU-3′<br>3′-TAACATGTGCCGGCTTA-5′	MZ-1480 and MZ-1455 annealed
MZ-1655	AUUGUACACGAAGGACUGAAU	Guide RNA oligonucleotide for smFRET measurements. Has a Cy3B donor dye on 5′-U21
MZ-1656	GTGGATGCGAACGATTGCTGTGAGAGATCT-Bio	Anchor oligonucleotide for smFRET measurements. Has an Atto647N acceptor dye on 5′-G1 and a biotin on 5′-T30
MZ-1715	AGATCTCTCACAGCAATCGTTCGCATCCACTTTTTTTT GAATTGAAGATGTGTACAACTTTT	8 bp-complementary target for smFRET measurements.
MZ-1745	AGGAGGGCGGAGCCUAUGGAAAAACGCCAC	30 nt RNA for cryo-EM duplex formation. Has 5′-P.
MZ-1747	GCCGCGCCGGTGGCGTTTTTCCATAGGCTCCGCCCTCC TGCCAGAGTTCGC	51 nt DNA target for cryo-EM duplex formation.
MZ-1752	AGATCTCTCACAGCAATCGTTCGCATCCACTTTTTTTT TTTTTTTTTTTTTTTTTTTTTTTT	Non-complementary target oligo for smFRET measurements

MZ-1800	AUUGUACACGGCCGAAU	17 nt RNA for cryo-EM duplex formation. Has 5′-P.
MZ-1897	CGGAATATATGTACAAA	17 nt DNA oligonucleotide used as competitor. Analogous to MZ-1560 but has a substitution t1 T→A
MZ-1898	CGGAATATATGTACAAC	17 nt DNA oligonucleotide used as competitor. Analogous to MZ-1560 but has a substitution t1 T→C
MZ-1899	CGGAATATATGTACAAG	17 nt DNA oligonucleotide used as competitor. Analogous to MZ-1560 but has a substitution t1 T→G
MZ-1900	CGGAATATATGTACATT	17 nt DNA oligonucleotide used as competitor. Analogous to MZ-1560 but has a substitution t2 A→T
MZ-1911	CGGAATATATGTACAGT	17 nt DNA oligonucleotide used as competitor. Analogous to MZ-1560 but has a substitution t2 A→G
MZ-1912	CGGAATATATGTACACT	17 nt DNA oligonucleotide used as competitor. Analogous to MZ-1560 but has a substitution t2 A→C
MZ-1901	CGGAATATATGTACTAT	17 nt DNA oligonucleotide used as competitor. Analogous to MZ-1560 but has a substitution t3 A→T
MZ-1913	CGGAATATATGTACGAT	17 nt DNA oligonucleotide used as competitor. Analogous to MZ-1560 but has a substitution t3 A→G
MZ-1914	CGGAATATATGTACCAT	17 nt DNA oligonucleotide used as competitor. Analogous to MZ-1560 but has a substitution t3 A→C
MZ-1902	CGGAATATATGTAGAAT	17 nt DNA oligonucleotide used as competitor. Analogous to MZ-1560 but has a substitution t4 C→G
MZ-1915	CGGAATATATGTAAAAT	17 nt DNA oligonucleotide used as competitor. Analogous to MZ-1560 but has a substitution t4 C→A
MZ-1916	CGGAATATATGTATAAT	17 nt DNA oligonucleotide used as competitor. Analogous to MZ-1560 but has a substitution t4 C→T

MZ-1903	CGGAATATATGTTCAAT	17 nt DNA oligonucleotide used as competitor. Analogous to MZ-1560 but has a substitution t5 A→T
MZ-1917	CGGAATATATGTGCAAT	17 nt DNA oligonucleotide used as competitor. Analogous to MZ-1560 but has a substitution t5 A→G
MZ-1918	CGGAATATATGTCCAAT	17 nt DNA oligonucleotide used as competitor. Analogous to MZ-1560 but has a substitution t5 A→C
MZ-1904	CGGAATATATGAACAAT	17 nt DNA oligonucleotide used as competitor. Analogous to MZ-1560 but has a substitution t6 T→A
MZ-1905	CGGAATATATCTACAAT	17 nt DNA oligonucleotide used as competitor. Analogous to MZ-1560 but has a substitution t7 G→C
MZ-1906	CGGAATATAAGTACAAT	17 nt DNA oligonucleotide used as competitor. Analogous to MZ-1560 but has a substitution t8 T→A

Supplementary Table S2. Strains, plasmids, and proteins used in this work.

Bacterial strains<br><br>(Escherichia coli)	Details	Source or reference, links
BL21(DE3)	F^- ompT gal dcm lon hsdS$_B$($r_B^-m_B^-$) λ(DE3 [lacI lacUV5-T7p07 ind1 sam7 nin5]) [malB$^+$]$_{K-12}$(λ^S)	Studier&Moffatt 1986, J. Mol. Biol. https://doi.org/10.1016/0022-2836(86)90385-2
Plasmids		
pETDuet_AfAgo-N	Bacterial expression vector with His$_6$-TEV-AfAgo-N gene.	https://benchling.com/s/seq-Kyx29GowMA91jJ3yW5fQ?m=slm-bFwXuxpZt21RHU1h4zSD
pETDuet_AfAgo	Bacterial expression vector with AfAgo gene.	https://benchling.com/s/seq-qyb3cWqmCKX4AYIX4sXR?m=slm-xdFtrcDOClc5BXTwpqmX
pBAD_fAfAgo	Bacterial expression vector with His$_6$-AfAgo-N and AfAgo genes.	https://benchling.com/s/seq-ddaL2rIgYyQdokySjPNy?m=slm-ghmmn1JkAYJFlkMGzTVI
pBAD_scfAfAgo	Bacterial expression vector with a scfAfAgo gene.	https://benchling.com/s/seq-jPXbDQzUSWtNLzKjIwci?m=slm-BKFbZayPJYLuIRNUAjcd
pBAD_TwinStrep-scfAfAgo	Bacterial expression vector with a TwinStrep-scfAfAgo gene.	https://benchling.com/s/seq-MmH81sIO3gB25ULKIPGq?m=slm-FyUrrhY5gZpvZ85hSZG7
pBAD_TwinStrep-AfAgo	Bacterial expression vector with a TwinStrep-AfAgo gene.	https://benchling.com/s/seq-NM4ng3rwgUGk5CyapymR?m=slm-boVvzF7S2Mz07qirUlBD
pCDFDuet_His-AfAgo-N	Bacterial expression vector with a His$_6$-AfAgo-N gene.	https://benchling.com/s/seq-KmezzqulpPWLyKvZ0STd?m=slm-czNO03DmyJ4Kv346IeBA

Supplementary Table S3. Crystal data collection and refinement statistics.

Structure	AfAgo-N 1.9 Å	AfAgo-N 1.4 Å	fAfAgo-DNA
Crystallization reservoir solution	Natrix2 #21 ("Hampton Research"): NaCl 12 mM, KCl 8 mM, sodium cacodylate pH 6.0, MPD 50% (v/v), spermine 12 mM	Tris-HCl pH 8.0 50 mM, Bicine pH 9.0 50 mM, 2-propanol 27% (v/v), ammonium acetate 110 mM	NaHepes pH 7.5 100 mM, Ethylene glycol 2% (v/v), PEG3350 10% (w/v)
Cryo protection solution	none	Ammonium acetate 160 mM, Tris-HCl pH 8.5 80 mM, 2-propanol 24% (v/v), glycerol 20% (v/v)	Reservoir solution supplemented with ethylene glycol to 30% (v/v)
Data collection statistics			
Space group	P 1	P 3_2 2 1	P 2_1 2_1 2_1
Cell constants	a=42.03 Å, b=57.84 Å, c=61.62 Å, α=73.94°, β=89.56°, γ=89.75°	a=75.26 Å, b=75.26 Å, c=94.72 Å, α=β=90°, γ=120°	a=81.71, b=105.89, c=144.35 α=β=γ =90°
Wavelength, Å	0.9768	0.9797	1.0100
PETRA III, EMBL C/O DESY beam line	P14	P13	P14
Unique reflections: overall (outer shell)	44415 (6503)	61624 (3045)	44100 (4576)
Resolution range, Å	55.59-1.95	94.72-1.40	144.35-2.59
Completeness: overall (outer shell), %	93.2 (93.5)	100 (100)	99.9 (100)
Multiplicity: overall (outer shell)	3.6 (3.6)	9.7 (8.4)	13.3 (13.7)
I/σ: overall (outer shell)	15.9 (2.2)	23.8 (2.3)	30.2 (6.6)
Rmerge: overall (outer shell), %	0.033 (0.492)	0.047 (0.908)	0.054 (0.413)
B-factor from Wilson, $Å^2$	31.8	18.7	65

Refinement statistics			
Reflections: work (non-anomalous)/test	39974 / 4433	55371 / 6208	39581 / 4454
Atom number: protein / DNA / solvent	3800 / - / 308	2116 / 373	5970 / 482 / 268
Rcryst (Rfree), %	0.17 / 0.22	0.18 / 0.21	0.17 / 0.23
RMSD: bond lengths, Å / bond angles, (°)	0.007 / 1.509	0.012 / 1.895	0.017 / 2.520
Ramachandran: favoured/allowed/ outliers, %	97% / 3% / 0%	98% / 2% / 0%	96% / 4% / 0%
average B-factors: all atoms/ main chain/ side chain/ DNA / solvent, $Å^2$	37.0 / 33.1 / 39.2 / -/42.3	24.0 / 18.9 / 23.5 / - / 37.7	60.0 / 54.5 / 57.1 / 70.2 / 62.4
PDB ID	8old	8olj	8ok9

Supplementary Table S4. Cryo-EM data collection and refinement statistics.

Complex	fAfAgo-g30/t51 heteroduplex	fAfAgo-g17/t17 heteroduplex
Data collection and processing		
Magnification	92000	92000
Voltage (kV)	200	200
Electron exposure (e^{-}/Å^2)	31	31
Defocus range (μm)	-1/-2	-1/-2
Pixel size (Å)	1.1	1.1
Symmetry imposed	C1	C1
No. of initial particle images	2 326 555	1 480 423
No. of final particle images	498 039	364 005
Map resolution (Å)	2.83	3.54
Map resolution FSC threshold (Å)	0.143	0.143
Map resolution range (Å)	2.428 - 46.575	2.378 - 46.009
Refinement		
Initial model used (PDB code)	8ok9	8ok9
Model resolution FSC threshold (Å)	0.143	0.143
Model resolution (Å)	2.8	3.54
Model composition: Non-hydrogen atoms / Protein/nucleic acid / Solvent	6544 / 5391 / 1111 / 42	6080 / 5372 / 708 / 0
R.m.s. d. from ideality: Bonds (Å)/Angles (°)	0.004 / 0.637	0.004 / 0.678
MolProbity score	1.79	2.24
Clashscore	13.73	20.14
Poor rotamers (%)	0.17	0.66
Ramachandran plot: Favored (%) / Allowed (%) / Disallowed (%)	97.24 / 2.76 / 0	93.25 / 6.75 / 0
Model B-factors: main chain (Å^2) / side chain (Å^2)	47.9 / 53.0	48.1 / 47.5
PDB ID	8pvv	8qg0
EMDB ID	EMD-17973	EMD-18386

Supplementary Table S5. Single-molecule FRET data. % interacting – percentage of detected traces with high acceptor intensity. Fraction high E – fraction of time with high acceptor intensity, calculated from all registered trajectories. fAfAgo-gRNA and scfAfAgo-gRNA display heightened FRET efficiencies and longer FRET durations with the 8 bp complementary target, compared to the non-complementary target and to gRNA alone, hinting at RNA-guided DNA targeting. RsAgo, used as a control, displays no such activity under conditions tested.

Sample	% interacting	Fraction high E
AfAgo-gRNA 1 nM with 8 bp complementary target	6	0.027
AfAgo-gRNA 10 nM with 8 bp complementary target	34	0.110
AfAgo-gRNA 1 nM with non-complementary target	1	0.005
AfAgo-gRNA 10 nM with non-complementary target	8	0.021
fAfAgo-gRNA 1 nM with 8 bp complementary target	28	0.066
fAfAgo-gRNA 10 nM with 8 bp complementary target	52	0.107
fAfAgo-gRNA 1 nM with non-complementary target	8	0.003
fAfAgo-gRNA 10 nM with non-complementary target	11	0.016
scfAfAgo-gRNA 1 nM with 8 bp complementary target	37	0.283
scfAfAgo-gRNA 10 nM with 8 bp complementary target	49	0.375
scfAfAgo-gRNA 1 nM with non-complementary target	15	0.016
scfAfAgo-gRNA 10 nM with non-complementary target	22	0.089
gRNA 10 nM with 8 bp complementary target	0	0.006
RsAgo-gRNA 50 nM with 8 bp complementary target	-	-

Supplementary Table S6. Alternative scenarios for pAgo evolution and steps needed to explain the observed data for each scenario.

Feature	Observation	Steps needed to explain the observed data	
		Long-A → long-B/short pAgos	Long-B/short → long-A pAgos
PIWI domain	Long-B/short pAgos have a catalytically inactive PIWI domain;<br>Some groups of long-A pAgos and eAgos also have an inactive PIWI domain.	Inactivation of the active site tetrad (DEDX, where X is D, H, or K) in some members leading to groups of inactive PIWI in some long-A pAgos and eAgos and all long-B/short pAgos.<br>**One-directional process**	Emergence of the DEDX tetrad from inactive PIWI and subsequent loss of the tetrad in some groups of long-A pAgos and eAgos.<br>**Requires action of two opposite processes**
PAZ domain	Short pAgos do not have a PAZ domain<br>Long-B pAgos are either PAZ-less or have a PAZ domain of various levels of reduction.	Random loss of PAZ structural elements or of the entire PAZ domain following inactivation of PIWI and diminished need for binding the 3′-end of a guide.<br>**Common process when domain function is no longer needed**	Building up the PAZ domain from scratch by adding/retaining only PAZ structural motifs until canonical PAZ domain structure is achieved.<br>**Highly unlikely process given random nature of evolutionary events**
Association with toxic effectors	In short pAgos, subunits representing the N-terminal lobe of Ago are fused with diverse effector domains;<br>long-B pAgos are accompanied by effector proteins;<br>long-A pAgos and eAgos are neither fused to, nor accompanied by toxic effectors.	Following PIWI inactivation and reduction or loss of PAZ domain, new pAgo molecular functions emerge through association with random toxic effector proteins either as a standalone partner or as a domain fusion.<br>**A common way to gain a new function**	Associated or fused toxic effector is lost and PAZ-less pAgo having inactive PIWI is on the route through random events to become an active long-A pAgo.<br>**Loss of associated or fused effectors is common, but activating PIWI and especially building up PAZ domain is unlikely (see above)**

Supplementary figures

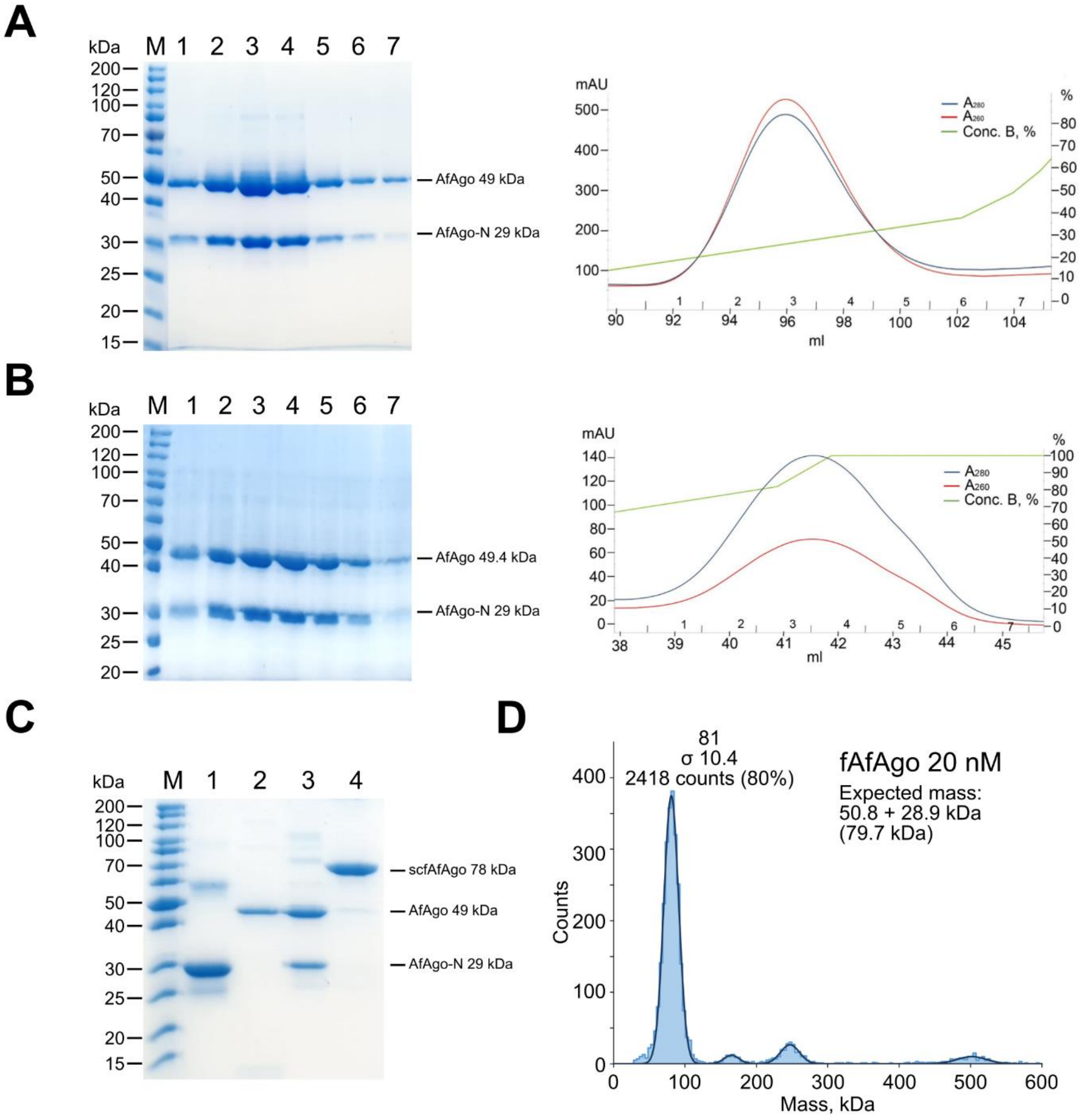

Supplementary Figure S1. (**A**) SDS-PAGE of fractions collected (left) after purification of fAfAgo by chromatography through HisTrap HP chelating column; numbering corresponds to numbers of the collected fractions indicated below the curves in the chromatogram (right). (**B**) SDS-PAGE of fractions collected after purification of fAfAgo by chromatography through HiTrap Heparin HP column (left); numbering corresponds to numbers of the collected fractions are indicated below the curves in the chromatogram (right). (**C**) SDS-PAGE of purified proteins: M – marker, 1 – the full-length upstream protein (AfAgo-N), 2 – AfAgo, 3 – the heterodimeric complex of AfAgo-N and AfAgo (fAfAgo), 4 – single chain fAfAgo (scfAfAgo). (**D**) Mass photometry histograms of fAfAgo protein. fAfAgo exhibits a

clear distribution centered around 81 kDa (80% of particles), which is close to the theoretical M_W of the heterodimeric complex.

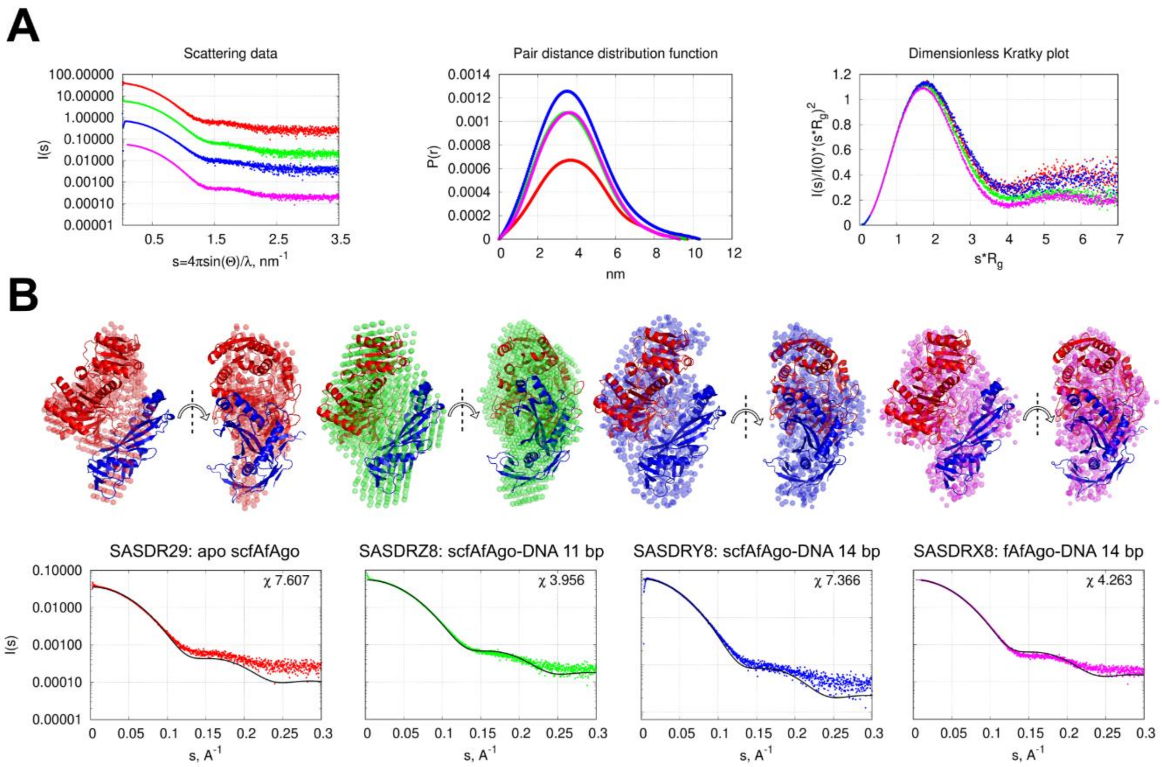

Supplementary Figure S2. (**A**) SAXS data of scfAfAgo (apo – red, SASBDB ID: SASDR29), complex with 11 bp DNA – green (SASBDB ID: SASDRZ8), complex with 14 bp DNA – blue (SASBDB ID: SASDRY8) and SEC-SAXS data of fAfAgo complex with 14 bp DNA – magenta (SASBDB ID: SASDRX8). Scattering curves are displaced along *y* axis for clarity. (**B**) Top: Shape reconstructions superimposed with protein chains of crystal structure fAfAgo with DNA. AfAgo subunit colored red, AfAgo-N – blue. Bottom: comparison of SAXS data with scattering function of fAfAgo complex calculated by CRYSOL (black curve). DNA was excluded from calculation. SASBDB IDs of scfAfAgo and fAfAgo complexes are indicated. SAXS experimental data colored as indicated in (**A**).

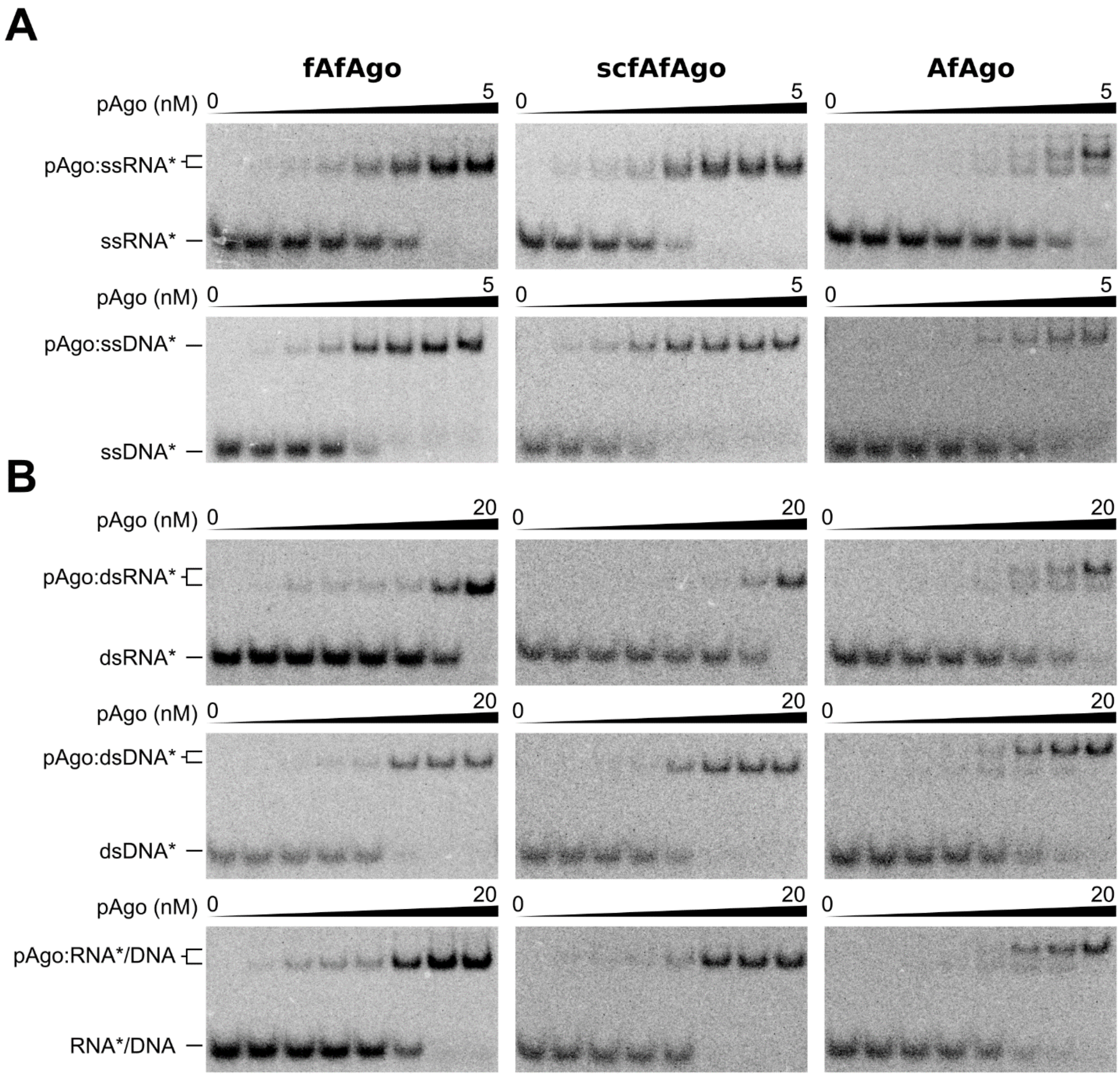

Supplementary Figure S3. EMSA results. (**A**) Single-stranded substrate (ssRNA and ssDNA) binding by fAfAgo, scfAfAgo and AfAgo. Protein concentrations in lanes: 0, 0.05, 0.1, 0.2, 0.5, 1, 2, 5 nM. (**B**) Double-stranded substrate (dsRNA, dsDNA and RNA/DNA) binding by fAfAgo, scfAfAgo and AfAgo. Protein concentrations in lanes: 0, 0.1, 0.5, 1, 2, 5, 10, 20 nM. Nucleic acid binding by AfAgo results in two binary complex populations, possibly due to homodimerization.

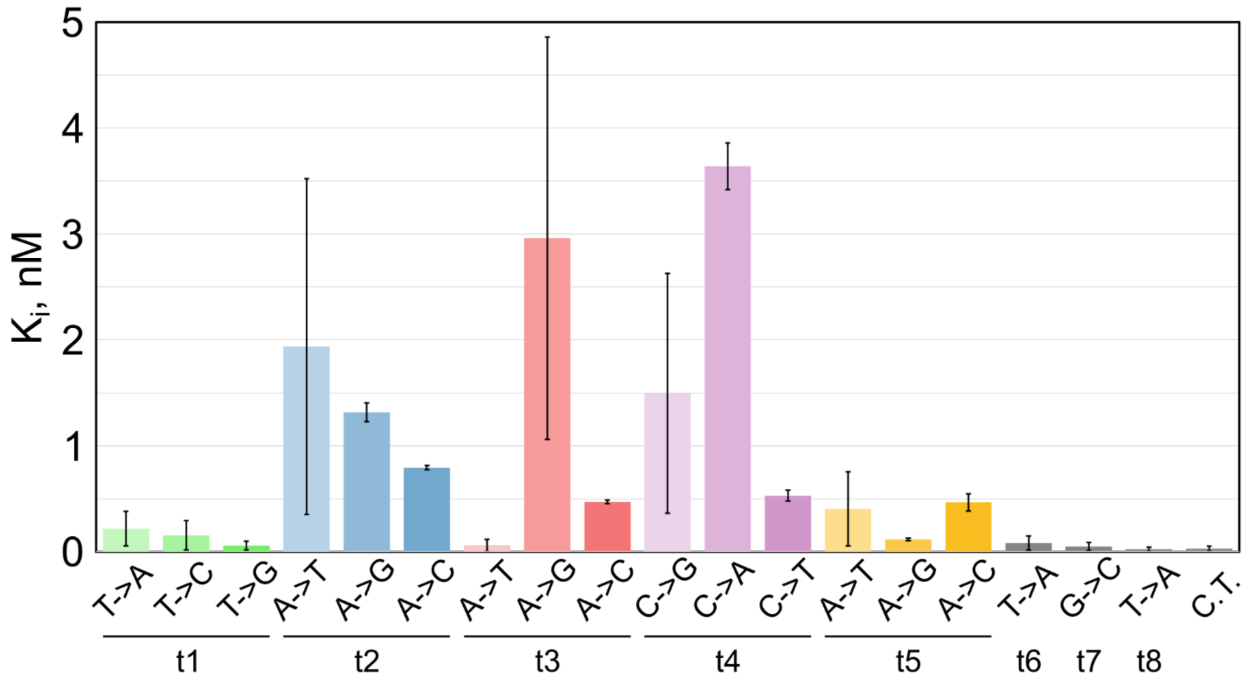

Supplementary Figure S4. Average inhibition constants K_i of various tDNA mismatches in competition with optimal tDNA. Number next to “t” indicates number of the nucleotide from target 3′-end, letters under each bar indicate the change made from optimal sequence. C.T. – complementary target control (self-competition). Bars are arithmetic means of at least two independent replicates, with error bars indicating s.d.

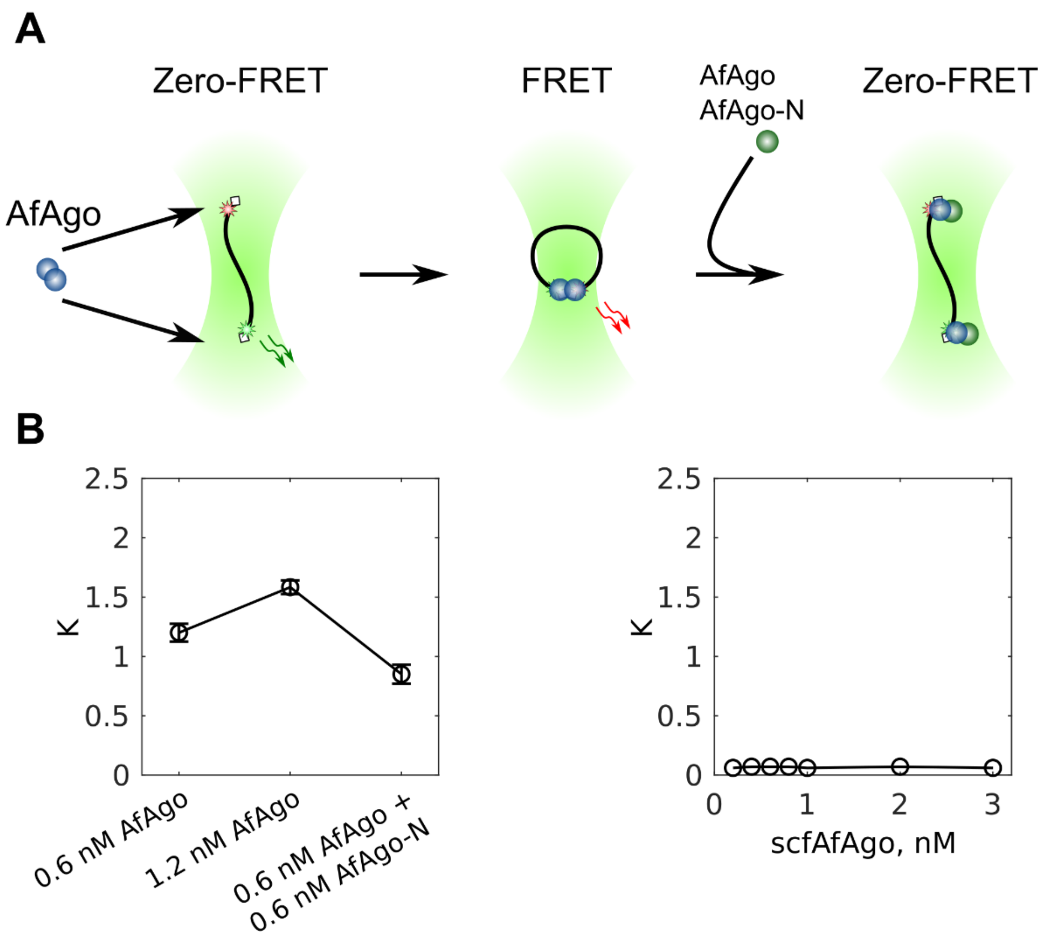

Supplementary Figure S5. Competitive single-molecule FRET results with dsDNA in solution. (**A**) Schematic representation of suggested protein-DNA interactions. (**B**) Supplementing 0.6 nM of AfAgo-N to high-efficiency FRET reaction (DNA + 0.6 nM AfAgo) lowers FRET efficiency, presumably by disrupting the looped AfAgo-DNA complex formed by AfAgo dimerization (left). scfAfAgo does not form dimers and, therefore, looped dsDNA complexes (right).

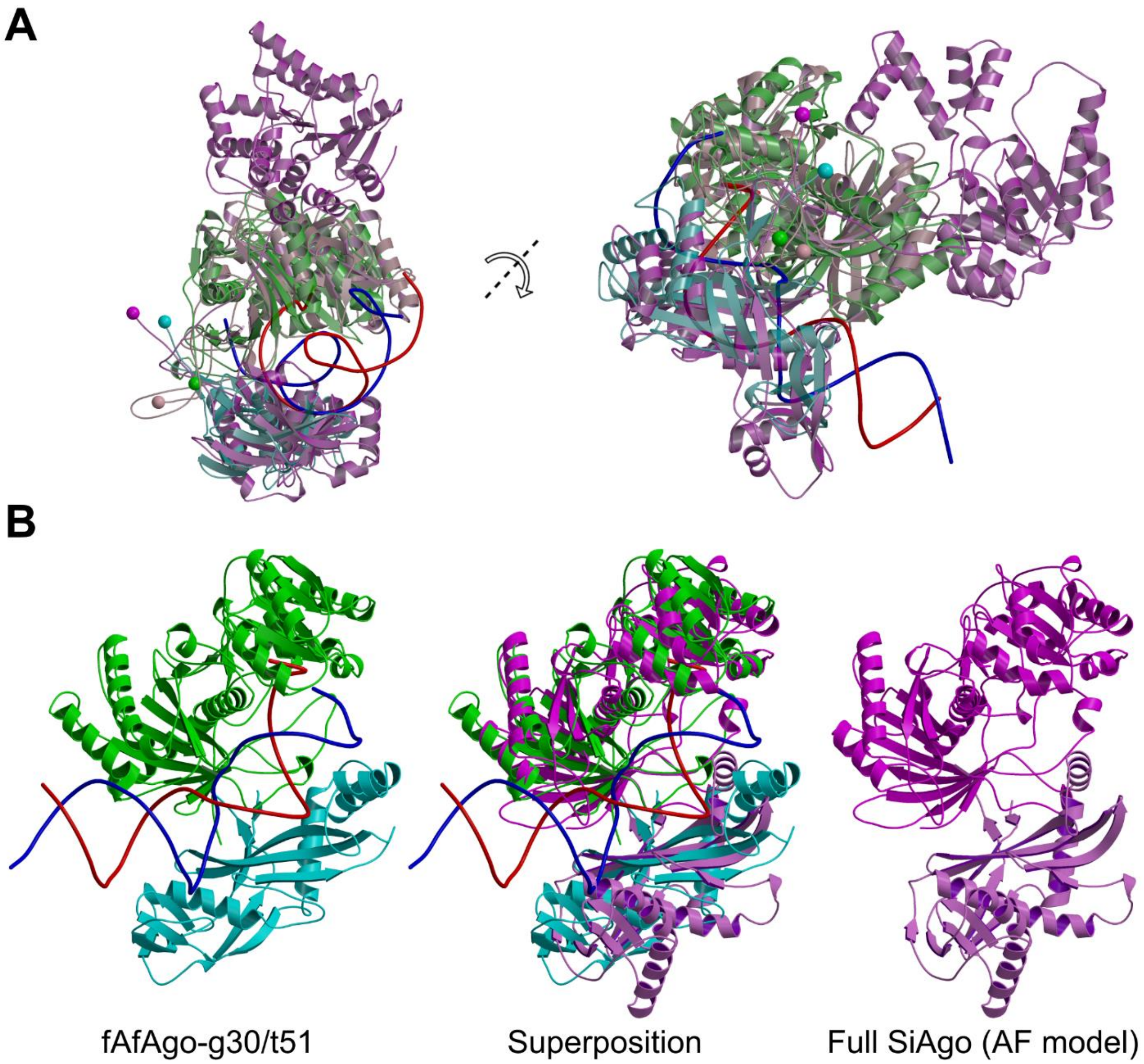

Supplementary Figure S6. (**A**) N- and C-termini of both proteins in fAfAgo complex are located close to each other. A similar situation was observed in the AlphaFold model of GsSir2/Ago (9). GsSir2/Ago is colored magenta (SIR2-APAZ protein) and green (Ago protein). The N-terminal residue of GsAgo is shown as a pink sphere, magenta sphere corresponds to the C-terminus of the GsSIR2-APAZ protein. The distance between these residues is 30 Å. It should be noted that the N-terminal part of the GsAgo protein is modeled in an elongated conformation, whereas in the real protein it could be folded in another way. In the fAfAgo complex, AfAgo protein is colored green, the N-terminal residue of AfAgo is indicated by the green sphere. Ago-N protein is colored cyan, its C-terminal atom is shown as a cyan sphere. The distance between these two points is 18 Å but the last 5 residues are not visible in the electron density. (**B**) Comparison of full AfAgo bound to DNA with AlphaFold model of full SiAgo (SiAgo-Aga1 heterodimer). N-terminal region is colored light blue, MID-PIWI, green. Structural similarity between full AfAgo and full SiAgo based on Dali comparison of individual chains:

N-lobe: 243 residue pairs superimposed, resulting RMSD=4.8 Å, sequence identity=14%

MID-PIWI: 459 residue pairs superimposed, resulting RMSD=2.9 Å, sequence identity=16%.

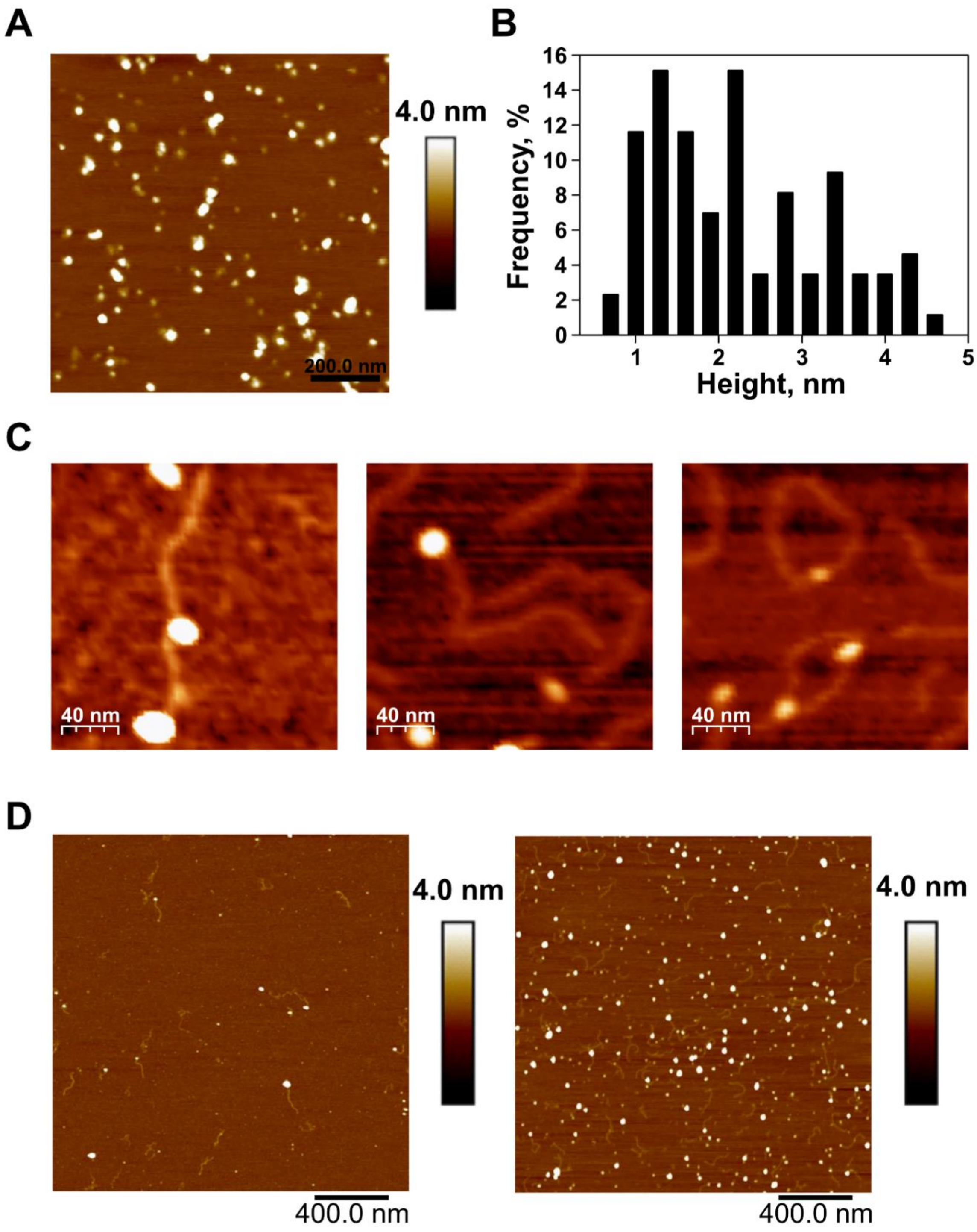

Supplementary Figure S7. Representative AFM topography image (**A**) and height distribution (**B**) of unbound fAfAgo protein complexes (n = 86) adsorbed on freshly cleaved mica. (**C**) Representative images of protein complex:DNA structures (from left to right): fAfAgo bound on random DNA strand locations; fAfAgo bound to the end of DNA strand; ring-shaped structures. (**D**) AFM topography image of adsorbed fAfAgo:DNA complexes on APS-modified mica prepared by quenching (left) and not quenching (right) the crosslinker. Area of interest 2 μm × 2 μm.

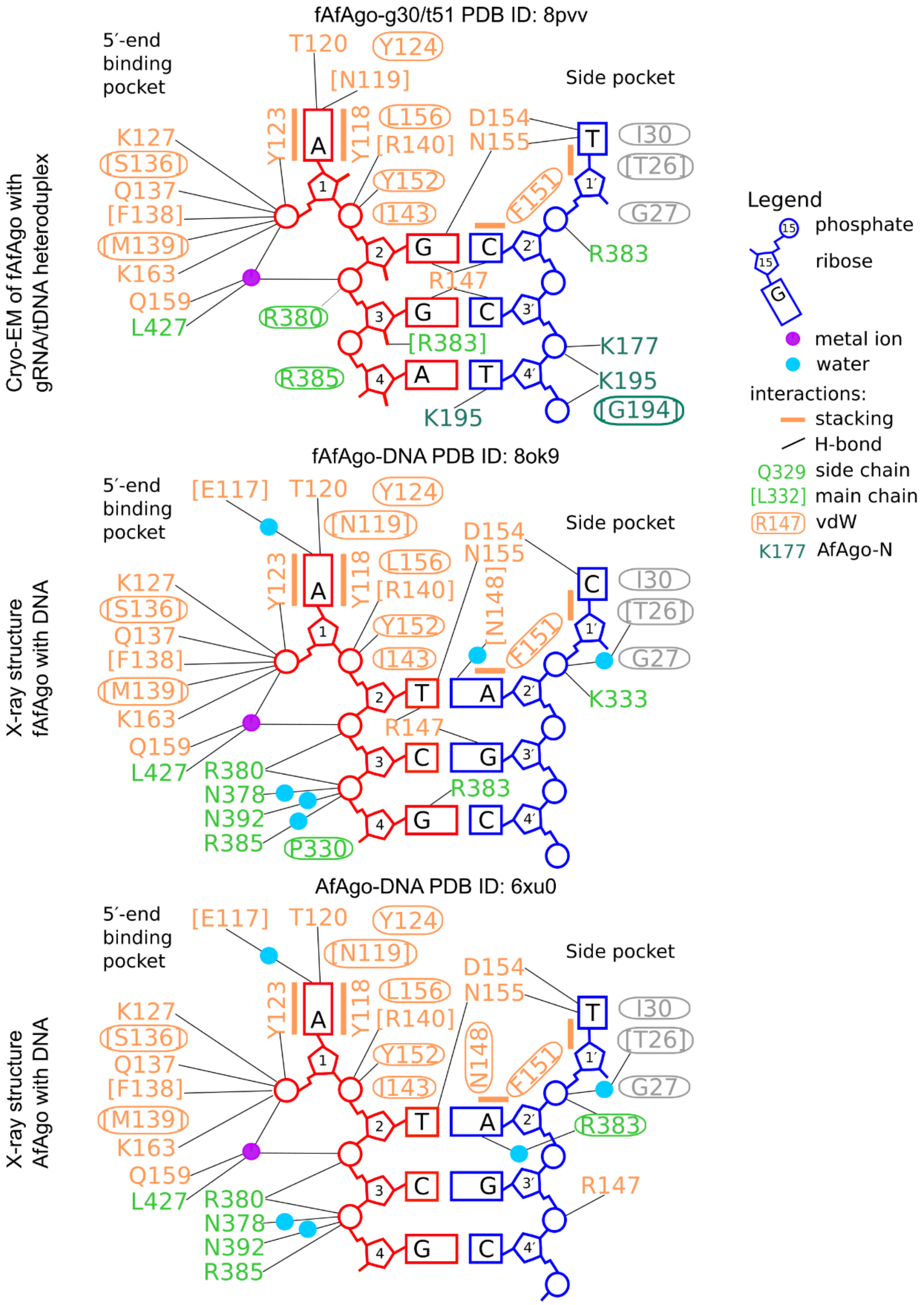

Supplementary Figure S8. Schematic depiction of conservative contacts of fAfAgo and AfAgo with 5′-end of the guide (red) and 3′-end of the target (blue) strands. Residues are color-coded as in Figure 5A: L1 – yellow; L2 – gray; MID –

orange; PIWI – green. The 5′-end base of the guide is displaced into a binding pocket. Three guide-target base pairs form short duplex in the crystal structure. Only four 5′-end of the guide and 3'-target strands are shown.

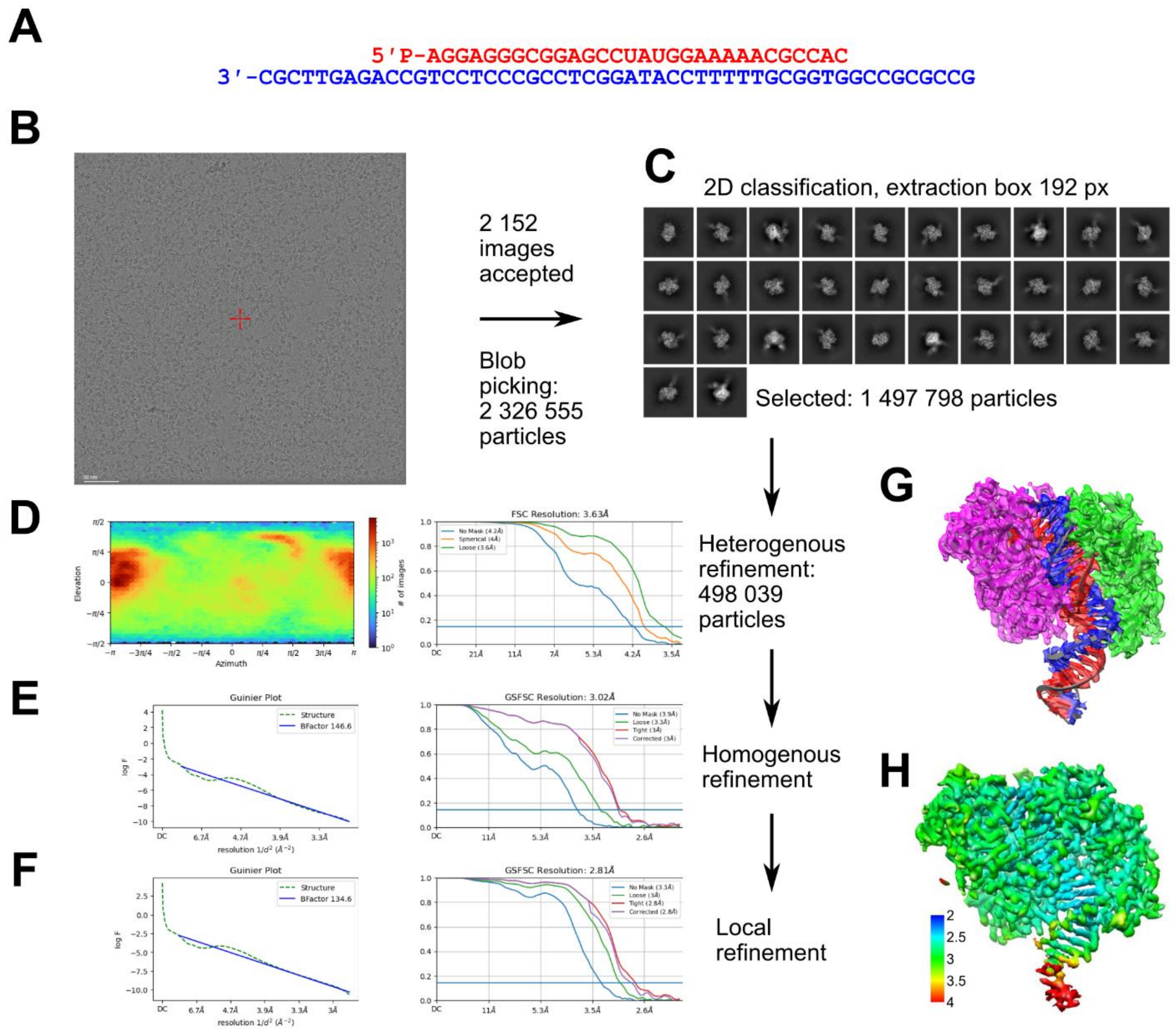

Supplementary Figure S9. fAfAgo complex with 30 nt 5′P-gRNA (red)/51 nt tDNA (blue) heteroduplex. Schematic representation of Cryo-EM structure solution. (**A**) Sequence of the heteroduplex. (**B**) Representative micrograph. (**C**) 2D classes selected after a few cycles of 2D classification. (**D**) The spatial distribution of particles and FSC plot of the best class after heterogenous refinement. (**E**) FSC and Guinier plot of the homogenous refinement step. (**F**) FSC and Guinier plot of the final refinement step – local refinement with mask. (**G**) The volume is colored according to the corresponding subunits: AfAgo – magenta, AfAgo-N – green, guide RNA strand is colored red and target DNA – blue. (**H**) Local resolution analysis of the refined volume.

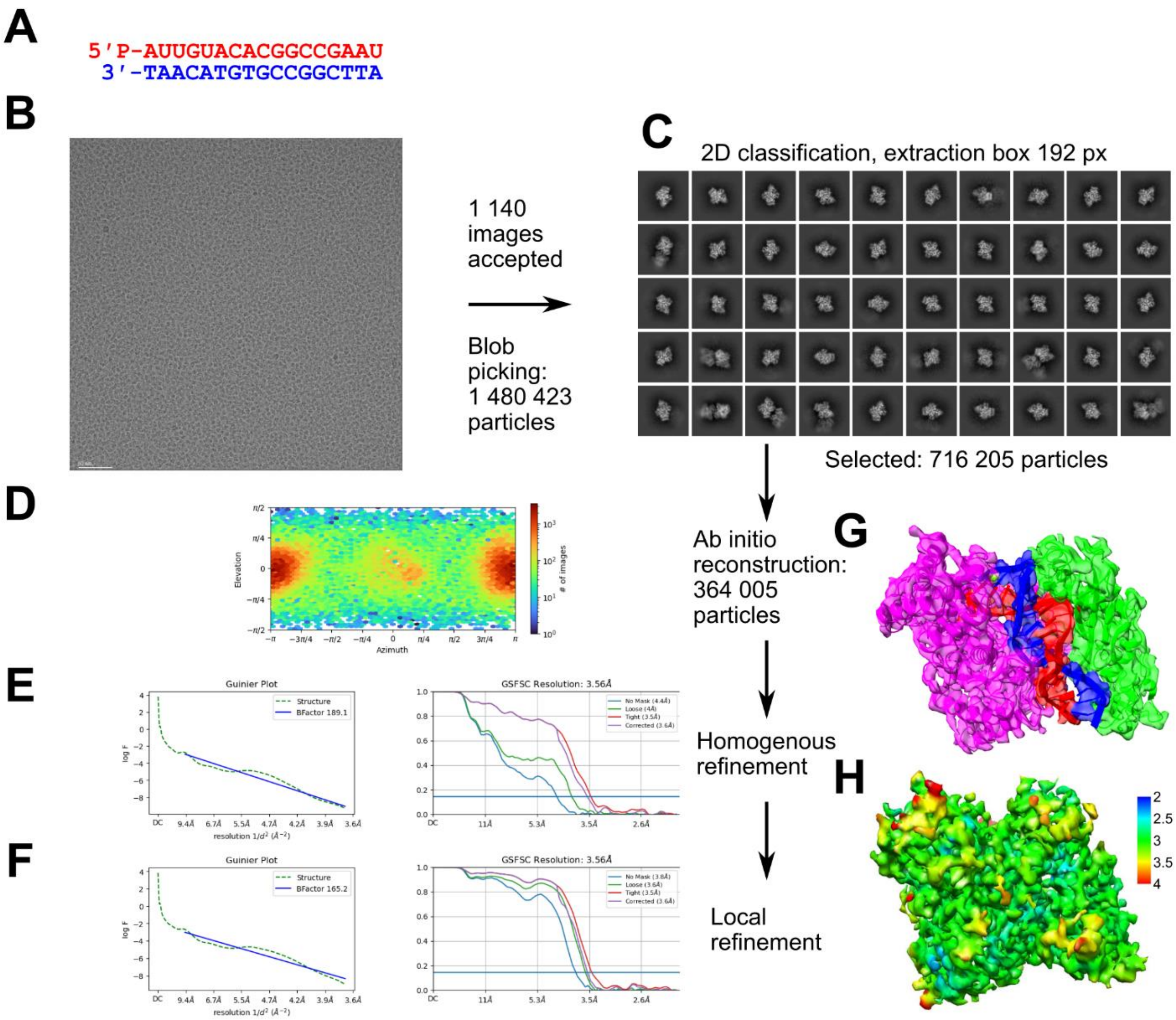

Supplementary Figure S10. fAfAgo complex with 17 bp 5′P-gRNA (red)/tDNA (blue) heteroduplex. Schematic representation of Cryo-EM structure solution. (**A**) Sequence of the heteroduplex. (**B**) Representative micrograph. (**C**) 2D classes selected after a few cycles of 2D classification. (**D**) The spatial distribution of particles of the *ab initio* 2D reconstruction is shown. (**E**) FSC and Guinier plots of homogenous refinement are shown. (**F**) FSC and Guinier plot of the final refinement step – local refinement – are shown. (**G**) The refined volume is colored according to the corresponding subunits: AfAgo – magenta, AfAgo-N – green, guide RNA strand is colored red and target DNA – blue. FSC and Guinier plot of the final refinement step are shown. (**H**) The final refined 3D volume is colored according to the local resolution.

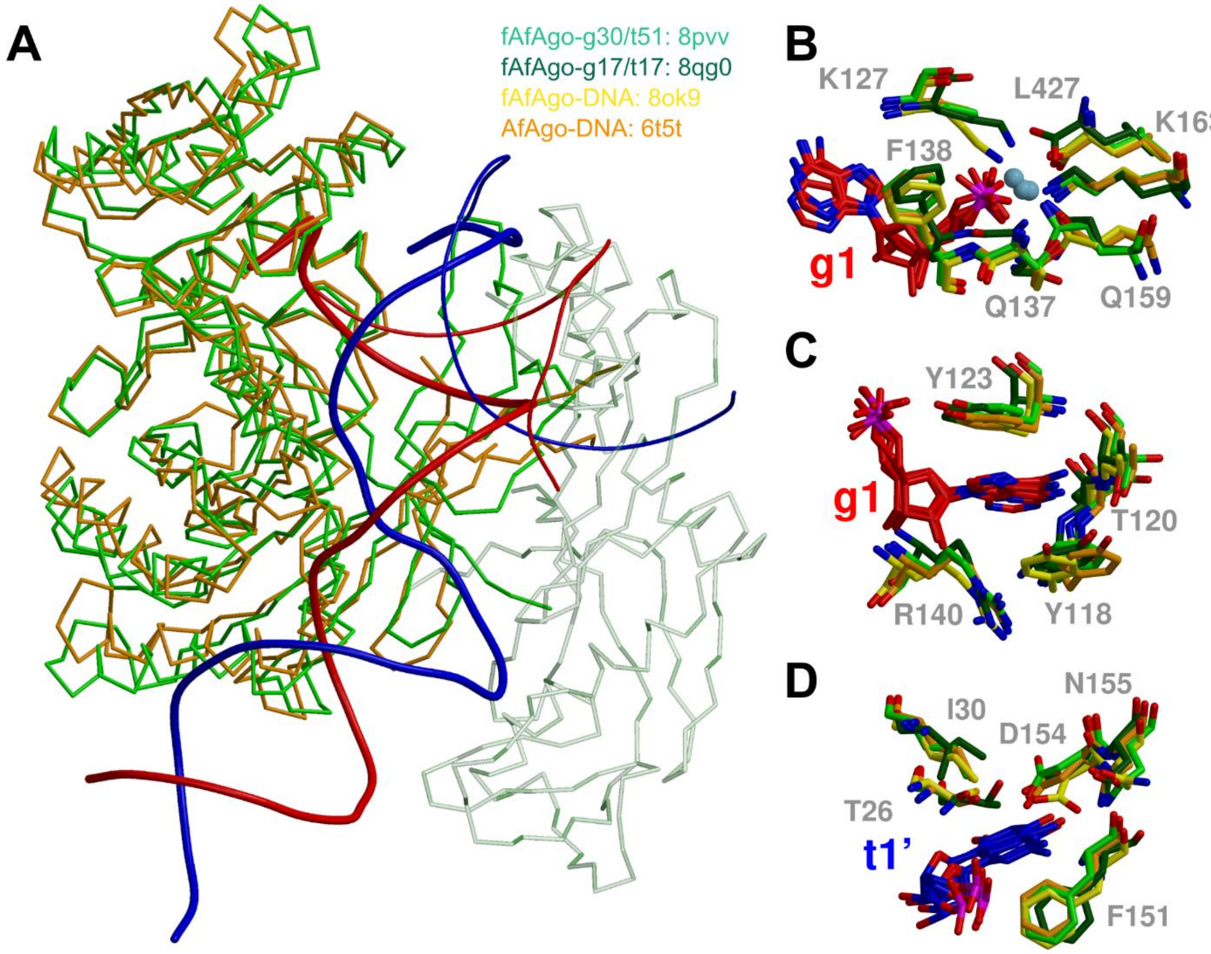

Supplementary Figure S11. (**A**) Comparison of fAfAgo-g30/t51 cryo-EM structure (green trace, AfAgo-N protein is transparent) and AfAgo crystal structure with dsDNA (PDB ID: 6t5t, orange trace). Guide strands are red, target - blue. Heteroduplex g30/t51 is shown as thick trace and dsDNA from 6t5t is shown as thin trace. (**B**) The binding of 5′-phosphate of the guide strand by fAfAgo complexes with nucleic acids and AfAgo-DNA. Mg^{2+} ion is shown as blue sphere. PDB IDs and color coding are shown in the legend. (**C**) Residues forming the first guide nucleotide binding pocket. (**D**) The binding of the first target nucleotide in the “side pocket”.

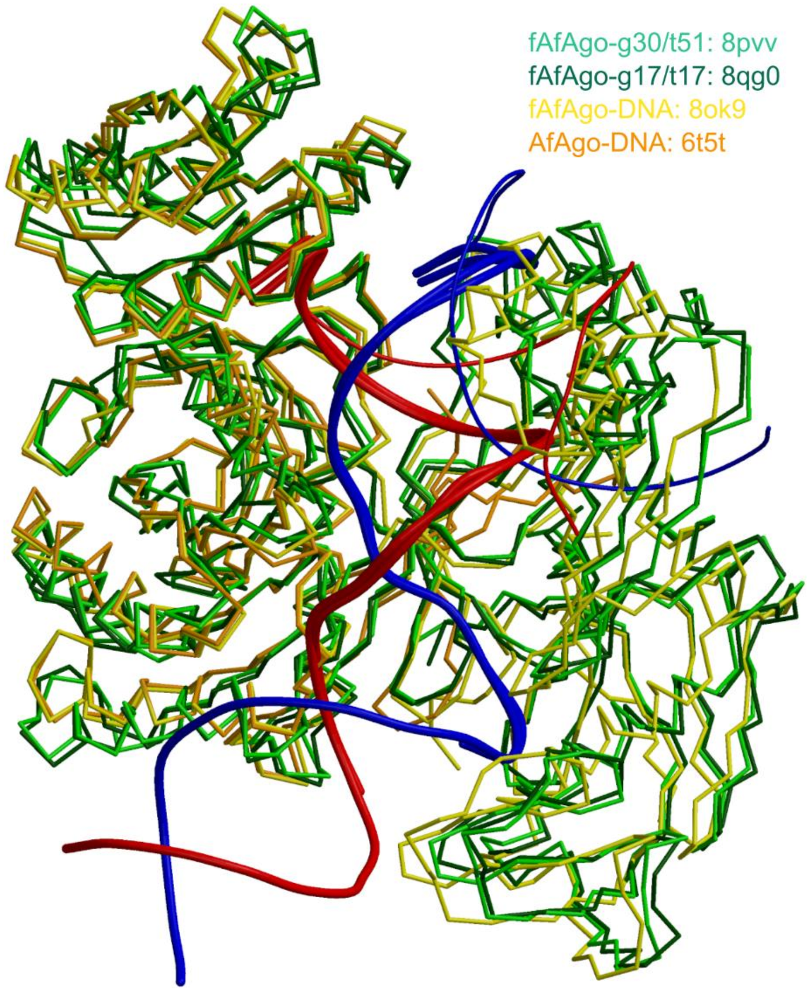

Supplementary Figure S12. Comparison of fAfAgo structures in complex with nucleic acids. Color coding of protein traces and PDB IDs are shown in the legend. DNA bound in the crystal structure of fAfAgo complex with DNA (yellow trace, PDB ID: 8ok9) is omitted for clarity. Heteroduplexes bound in cryo-EM structures: fAfAgo-g30/t51 (green trace, PDB ID: 8pvv) and fAfAgo-g17/t17 (dark green trace, PDB ID: 8qg0) are shown as thick traces. DNA oligoduplex in AfAgo homodimer crystal structure (orange trace, PDB ID: 6t5t) is shown as a thin trace. Guide strands are red, target – blue. RMSD between both superimposed Cryo-EM structures is 1.2 Å. Crystal structures of AfAgo (PDB ID: 6t5t) and fAfAgo with DNA (PDB ID: 8ok9) superimposed with RMSD 1.7 Å. From the other hand, RMSD of superpositions Cryo-EM structures with guide-target heteroduplexes with DNA bound crystal structures are slightly larger: 1.9-2.4 Å, as calculated by PDBe Fold v2.59, 377 residues overlaid.
